# Sequential Monte Carlo with transformations

**DOI:** 10.1007/s11222-019-09903-y

**Published:** 2019-11-17

**Authors:** Richard G. Everitt, Richard Culliford, Felipe Medina-Aguayo, Daniel J. Wilson

**Affiliations:** 1 Department of Statistics, University of Warwick, Coventry, CV4 7AL UK; 2 Department of Mathematics and Statistics, University of Reading, Reading, UK; 3 Nuffield Department of Medicine, University of Oxford, Oxford, UK

**Keywords:** Bayesian model comparison, Coalescent, Trans-dimensional Monte Carlo

## Abstract

This paper examines methodology for performing Bayesian inference sequentially on a sequence of posteriors on spaces of different dimensions. For this, we use sequential Monte Carlo samplers, introducing the innovation of using deterministic transformations to move particles effectively between target distributions with different dimensions. This approach, combined with adaptive methods, yields an extremely flexible and general algorithm for Bayesian model comparison that is suitable for use in applications where the acceptance rate in reversible jump Markov chain Monte Carlo is low. We use this approach on model comparison for mixture models, and for inferring coalescent trees sequentially, as data arrives.

**Electronic supplementary material:**

The online version of this article (10.1007/s11222-019-09903-y) contains supplementary material, which is available to authorized users.

## Introduction

### Sequential inference

Much of the methodology for Bayesian computation is designed with the aim of approximating a posterior $\pi$. The most prominent approach is to use Markov chain Monte Carlo (MCMC), in which a Markov chain that has $\pi$ as its limiting distribution is simulated. It is well known that this process may be computationally expensive; that it is not straightforward to tune the method automatically; and that it can be challenging to determine how long to run the chain for. Therefore, designing and running an MCMC algorithm to sample from a particular target $\pi$ may require much human input and computer time. This creates particular problems if a user is in fact interested in a number of target distributions $\left( \pi _{t}\right) _{t=1}^{T}$ defined possibly on different spaces: using MCMC on each target requires additional computer time to run the separate algorithms and each may require human input to design the algorithm, determine the burn in, etc. This paper has as its subject the task of using a Monte Carlo method to simulate from each of the targets $\pi _{t}$ that avoids these disadvantages.

Particle filtering (Gordon et al. 1993) and its generalisation, the SMC sampler (Del Moral et al. 2006) is designed to tackle problems of this nature. Roughly speaking, the idea of these approaches is to begin by using importance sampling (IS) to find a set of weighted *particles* that give an empirical approximation to $\pi _{0}$ then to, for $t=0,...,T-1$, update the set of particles approximating $\pi _{t}$ such that they, after changing their positions using a kernel $K_{t+1}$ and updating their weights, approximate $\pi _{t+1}$. This approach is particularly useful where neighbouring target distributions in the sequence are similar to each other, and in this case has the following advantages over running *T* separate MCMC algorithms.

- The similarity of neighbouring targets can be exploited since particles approximating $\pi _{t}$ may not need much adjustment to provide a good approximation to $\pi _{t+1}$. We have the desirable property that we find approximations to each of the targets in the sequence. Further, we also may gain when compared to running a single MCMC algorithm to target $\pi _{T}$, since it may be complicated to set up an MCMC that simulates well from $\pi _{T}$ without using a sequence of simpler distributions to guide particles into the appropriate regions of the space.
- When the targets $\left( \pi _{t}\right) _{t=1}^{T}$ are only known up to a constant of proportionality, SMC samplers also provide unbiased estimates of the corresponding normalising constants. In a Bayesian context, the normalising constant of $\pi _{t}$ is the *marginal likelihood* or *evidence*, a key quantity in Bayesian model comparison. For much of the paper, and in abuse of notation, we use the same letters for denoting distributions and corresponding densities. In addition, we use tildes to denote unnormalised densities; e.g., let $\theta \sim \pi _t(\cdot )$ then its density is given by $\pi _t\left( \theta \right) ={\tilde{\pi }}_t\left( \theta \right) /Z_t$, where $Z_t$ denotes the normalising constant.

### Outline of paper

In this paper, we consider the case where each $\pi _{t}$ is defined on a space of different dimension, often of increasing dimension with *t*. We provide a general framework for implementing an SMC algorithm in the aforementioned setting. A particle filter is designed to be used in a special case of this situation: the case where $\pi _{t}$ is the path distribution in a state space model, $\pi _{t}\left( \theta _{1:t}{\mid } y_{1:t}\right)$. A particle filter exploits the Markov property in order to update a particle approximation of $\pi _{t}\left( \theta _{1:t}{\mid } y_{1:t}\right)$ to an approximation of $\pi _{t+1}\left( \theta _{1:t+1}{\mid } y_{1:t+1}\right)$. In this paper, we consider targets in which there is not such a straightforward relationship between $\pi _{t}$ and $\pi _{t+1}$. In addition, the approach we present is useful in Bayesian model comparison that results from constructing an SMC sampler where each $\pi _{t}$ corresponds to a different model and there are *T* models that can be ordered, usually in order of their complexity. Deterministic transformations are used to move points between one distribution and the next, potentially yielding efficient samplers by reducing the distance between successive distributions. We also show how the same framework can be used for sequential inference under the coalescent model (Kingman 1982).

The use of deterministic transformations to improve SMC has been considered previously in a number of papers (e.g., Chorin and Tu 2009; Vaikuntanathan and Jarzynski 2011; Reich 2013; Heng et al. 2015; South et al. 2019). Several of these papers are focussed on how to construct useful transformations in a generic way including, for example: methods that map high density regions of the proposal to high density regions of the target (Chorin and Tu 2009) and methods that approximate the solution of ordinary differential equations that mimic the SMC dynamics (Heng et al. 2015). This paper is different in that it focuses on the particular case of a sequence of distribution on spaces of different dimensions, and uses transformations and proposals that are designed for the applications we study.

Section 2 describes the methodology introduced in the paper, considering both practical and theoretical aspects, and provides comparison to existing methods. We provide an example of the use of the methodology for Bayesian model comparison in Sect. 3, on the Gaussian mixture model. In Sect. 4, we use our methodology for online inference under the coalescent, using the flexibility of our proposed approach to describe a method for moving between coalescent trees. In Sect. 5, we present a final discussion and outline possible extensions.

## SMC samplers with transformations

### SMC samplers with increasing dimension

The use of SMC samplers on a sequence of targets of increasing dimension has been described previously (e.g., Naesseth et al. 2014; Everitt et al. 2017; Dinh et al. 2018). These papers introduce an additional proposal distribution for the variables that are introduced at each step. In this section, we straightforwardly see that this is a particular case of the SMC sampler in Del Moral et al. (2007).

#### SMC samplers with MCMC moves

To introduce notation, we first consider the standard case in which the dimension is fixed across all iterations of the SMC. For simplicity, we consider only SMC samplers with MCMC moves, and we consider an SMC sampler that has *T* iterations. Let $\pi _{t}$ be our target distribution of interest at iteration *t*, this being the distribution of the random vector $\theta _{t}$ on space *E*. Throughout the paper, the values taken by particles in the SMC sampler have a $^{(p)}$ superscript to distinguish them from random vectors; so for example $\theta _{t}^{(p)}$ is the value taken by the *p*th particle. We define $\pi _{0}$ to be a distribution from which we can simulate directly, simulate each particle $\theta _{0}^{(p)}\sim \pi _{0}$ and set its normalised weight $w_{0}^{(p)}=1/P$. Then for $0\le t<T$ at the $\left( t+1\right)$th iteration of the SMC sampler, the following steps are performed.

1. **Reweight** Calculate the updated (unnormalised) weight ${\tilde{w}}_{t+1}^{(p)}$ of the *p*th particle
  1 $$\begin{aligned} {\tilde{w}}_{t+1}^{(p)}= & {} w_{t}^{(p)}\frac{{\tilde{\pi }}_{t+1}\left( \theta _{t}^{(p)}\right) }{{\tilde{\pi }}_{t}\left( \theta _{t}^{(p)}\right) }. \end{aligned}$$
2. **Resample** Normalise the weights to obtain normalised weights $w_{t+1}^{(p)}$ and calculate the *effective sample size* (ESS) (Kong et al. 1994). If the ESS falls below some threshold, e.g., $\alpha P$ where $0<\alpha <1$, then resample.
3. **Move** For each particle use an MCMC move with target $\pi _{t+1}$ to move $\theta _{t}^{(p)}$ to $\theta _{t+1}^{(p)}$.

We remark that the move step above does not necessarily imply using a single MCMC iteration; if the chosen MCMC mixes slowly then performing many iterations and using adaptive strategies will result beneficial. The previous algorithm yields an empirical approximation of $\pi _{t}$ and an estimate of its normalising constant $Z_t$

2 $$\begin{aligned} {\hat{\pi }}_{t}^{P}=\sum _{p=1}^{P}w_{t}^{(p)}\delta _{\theta _{t}^{(p)}},\quad {\widehat{Z}}_t=\prod _{s=0}^{t}\sum _{p=1}^{P}w_{s}^{(p)}\frac{{\tilde{\pi }}_{s+1}\left( \theta _{s}^{(p)}\right) }{{\tilde{\pi }}_{s}\left( \theta _{s}^{(p)}\right) } \end{aligned}$$

where $\delta _{\theta }$ is a Dirac mass at $\theta$.

#### Increasing dimension

We now describe a case where the parameter $\theta$ increases in dimension with the number of SMC iterations. Our approach is to set up an SMC sampler on an extended space that has the same dimension of the maximum dimension of $\theta$ that we will consider [similarly to Carlin and Chib (1995)]. At SMC iteration *t*, we use: $\theta _{t}$ to denote the random vector of interest; $u_{t}$ to denote a random vector that contains the additional dimensions added to the parameter space at iteration $t+1$, and $v_{t}$ to denote the remainder of the dimensions that will be required at future iterations. Our SMC sampler is constructed on a sequence of distributions $\varphi _{t}$ of the random vector $\vartheta _{t}=\left( \theta _{t},u_{t},v_{t}\right)$ in space $E=\left( \varTheta _t, U_t, V_t \right)$, with

3 $$\begin{aligned} \varphi _{t}\left( \vartheta _{t}\right) =\pi _{t}\left( \theta _{t}\right) \psi _{t}\left( u_{t}{\mid }\theta _{t}\right) \phi _{t}\left( v_{t}{\mid }\theta _{t},u_{t}\right) , \end{aligned}$$

where $\pi _{t}$ is the distribution of interest at iteration *t*, and $\psi _{t}$ and $\phi _{t}$ are (normalised) distributions on the additional variables so that $\pi _{t}$ and $\varphi _{t}$ have the same normalising constant. The weight update in this SMC sampler is

4 $$\begin{aligned} {\tilde{w}}_{t+1}^{(p)}=w_{t}^{(p)}\frac{{\tilde{\pi }}_{t+1}\left( \theta _{t}^{(p)},u_{t}^{(p)}\right) }{{\tilde{\pi }}_{t}\left( \theta _{t}^{(p)}\right) \psi _{t}\left( u_{t}^{(p)}{\mid }\theta _{t}^{(p)}\right) }. \end{aligned}$$

Here, as in particle filtering, by construction, the $\phi _{t}$ terms in the numerator and denominator have cancelled so that none of the dimensions added after iteration $t+1$ are involved; a characteristic shared by the MCMC move with target $\varphi _{t+1}$, that need only update $\theta _{t}$, $u_{t}$.

### Motivating example: Gaussian mixture models

#### RJMCMC for Gaussian mixture models

The following sections make use of transformations and other ideas in order to improve the efficiency of the sampler. To motivate this, we consider the case of Bayesian model comparison, in which the $\pi _{t}$ are different models ordered by their complexity. In Sect. 3, we present an application to Gaussian mixture models, and we use this as our motivating example here. Consider mixture models with *t* components, to be estimated from data *y*, consisting of *N* observed data points. For simplicity, we describe a “without completion” model, where we do not introduce a label *z* that assigns data points to components. Let the *s*th component have a mean $\mu _{s}$, precision $\tau _{s}$ and weight $\nu _{s}$, with the weights summing to one over the components. Let $p_{\mu }$ and $p_{\tau }$ be the respective priors on these parameters, which are the same for every component, and let $p_{\nu }$ be the joint prior over all of the weights. The likelihood under *t* components is

5 $$\begin{aligned} f_{t}\left( y{\mid }\theta _{t}=\left( \mu _{s},\tau _{s},\nu _{s}\right) _{s=1}^{t}\right) =\prod _{i=1}^{N}\sum _{s=1}^{t}\nu _{s}{\mathcal {N}}\left( y_{i}{\mid }\mu _{s},\tau _{s}^{-1}\right) .\nonumber \\ \end{aligned}$$

An established approach for estimating mixture models is that of RJMCMC. Here, *t* is chosen to be a random variable and assigned a prior $p_{t}$, which here we choose to be uniform over the values 1 to *T*. Let

6 $$\begin{aligned}&\pi _{t}\left( \theta _{t}\right) =\pi \left( \theta _{t}{\mid } t,y\right) \nonumber \\&\quad \propto p_{\nu }\left( \nu _{1:t}\right) \left( \prod _{s=1}^{t}p_{\mu } \left( \mu _{s}\right) p_{\tau }\left( \tau _{s}\right) \right) f_{t} \left( y{\mid }\theta _{t}=\left( \mu _{s},\tau _{s},\nu _{s}\right) _{s=1}^{t}\right) \end{aligned}$$

be the joint posterior distribution over the parameters $\theta _{t}$ conditional on *t*. RJMCMC simulates from the joint space of $\left( t,\theta _{t}\right)$ in which a mixture of moves is used, some fixed-dimensional (*t* fixed) and some trans-dimensional (to mix over *t*). The simplest type of trans-dimensional move in this case is that of a birth move for moving from *t* to $t+1$ components or a death move for moving from $t+1$ to *t* (Richardson and Green 1997). We consider a birth move, a uniform prior probability over *t* and equal probability of proposing birth or death. For the purposes of exposition, we assume that the weights of the components are chosen to be fixed in each model. (This assumption will be relaxed later in Sect. 3.) Let $u_{t}=\left( \mu _{t+1},\tau _{t+1}\right)$, be the mean and precision of the new component and let $\psi _{t}\left( u_{t}{\mid }\theta _{t}\right) =p_{\mu }\left( \mu _{t+1}\right) p_{\tau }\left( \tau _{t+1}\right)$. A birth move simulates $u_{t}\sim \psi _{t}$ and has acceptance probability

7 $$\begin{aligned} \alpha =\min \left\{ 1,\frac{\pi _{t+1}\left( \theta _{t+1}\right) }{\pi _{t}\left( \theta _{t}\right) \psi _{t}\left( u_{t} {\mid } \theta _t \right) }\right\} , \end{aligned}$$

where $$\theta _{t+1}=\left( \theta _t, u_t \right) $$.

#### Comparing RJMCMC and SMC samplers

Consider the use of an SMC sampler for inference where the sequence of target distributions is $\left( \pi _{t}\right) _{t=1}^{T}$, i.e., the *t*th distribution is the mixture of Gaussians with *t* components. By choosing $u_t$ and $\psi _t$ as above, together with

$$\begin{aligned} v_{t}=\left( \mu _{(t+2):T},\tau _{(t+2):T}\right) \end{aligned}$$

and

$$\begin{aligned} \phi _{t}\left( v_{t}{\mid }\theta _{t},u_{t}\right) =\prod _{s=t+2}^{T}p_{\mu }\left( \mu _{s}\right) p_{\tau }\left( \tau _{s}\right) , \end{aligned}$$

we may use the SMC sampler described in Sect. 2.1.2. Note that the ratio in the acceptance probability in Eq. (7) is the same as the incremental SMC weight in Eq. (4). The reason for this is that both algorithms make use of an IS estimator of the Bayes factor $Z_{t+1}/Z_{t}$: using a proposed point $\theta _{t}\sim \pi _{t}$, $u_{t}\sim \psi _{t}$ and $\theta _{t+1}=\left( \theta _{t},u_{t}\right)$, this estimator is given by

8 $$\begin{aligned} \widehat{\frac{Z_{t+1}}{Z_{t}}}=\frac{\pi _{t+1}\left( \theta _{t+1}\right) }{\pi _{t}\left( \theta _{t}\right) \psi _{t}\left( u_{t}{\mid }\theta _{t}\right) }. \end{aligned}$$

We may see RJMCMC as using an IS estimator of the ratio of the posterior model probabilities within its acceptance ratio; this view on RJMCMC (Karagiannis and Andrieu 2013) links it to pseudo-marginal approaches (Andrieu and Roberts 2009) in which IS estimators of target distributions are employed. As in pseudo-marginal MCMC, the efficiency of the chain depends on the variance of the estimator that is used. We observe that the IS estimator in Eq. (8) is likely to have high variance: this is one way of explaining the poor acceptance rate of dimension changing moves in RJMCMC. In particular, we note that this estimator suffers a curse of dimensionality in the dimension of $\theta _{t+1}$, meaning that RJMCMC is in practice seldom effective when the parameter space is of high dimension. This view suggests a number of potential improvements to RJMCMC with a birth move, each of which has been previously investigated.

- IS performs better if the proposal distribution is close to the target, whilst ensuring that the proposal has heavier tails than the target. The original RJMCMC algorithm allows the possibility to construct such proposals by allowing for the use of transformations to move from the parameters of one model to the parameters of another. Richardson and Green (1997) provide a famous example of this in the Gaussian mixture case in the form of split-merge moves. Focusing on the split move, the idea is to propose splitting an existing component, using a moment matching technique to ensure that the new components have appropriate means, variances and weights.
- Annealed importance sampling (AIS) (Neal 2001) yields a lower variance than IS. The idea is to use intermediate distributions to form a path between the IS proposal and target, using MCMC moves to move points along this path. This approach was shown to be beneficial in some cases by Karagiannis and Andrieu (2013).
- The estimator in Eq. (8) uses only a single importance point. It would be improved by using multiple points. However, using such an estimator directly within RJMCMC leads to a “noisy” algorithm that does not have the correct target distribution for the same reasons as those given for the noisy exchange algorithm in Alquier et al. (2016). We note that recent work (Andrieu et al. 2018) suggests a correction to provide an exact approach based on the same principle.

The approach we take in this paper is to investigate variations on these ideas within the SMC sampler context, rather than RJMCMC. We begin by examining the use of transformations in Sect. 2.3, then describe the use of intermediate distributions and other refinements in Sect. 2.4. The final idea is automatically used in the SMC context, due to the use of *P* particles.

### Using transformations in SMC samplers

We now show (in a generalisation of Sect. 2.1.2) how to use transformations within SMC, whilst simultaneously changing the dimension of the target at each iteration; an approach we will refer to as *transformation SMC *(TSMC). We again use the approach of performing SMC on a sequence of targets $\varphi _{t}$, with each of the these targets being on a space of fixed dimension, constructed such that they have the desired target $\pi _{t}$ as a marginal. In this section, the dimension of the space on which $\pi _{t}$ is defined again varies with *t*, but is not necessarily increasing with *t*. Let $\theta _{t}$ be the random vector of interest at SMC iteration *t*: we wish to approximate the distributions $\pi _{t}$ of $\theta _{t}$ in the space $\varTheta _{t}$. Let $\left( {\tilde{\varphi }}_{t} \right) _{t=1}^T$ be a sequence of unnormalised targets, whose normalised versions are $\left( \varphi _{t} \right) _{t=1}^T$ and being the distribution of the random vector $\vartheta _{t}=\left( \theta _{t},u_{t}\right)$ in the space $E_{t}=\left( \varTheta _{t},U_{t}\right)$ where

$$\begin{aligned} {\tilde{\varphi }}_{t}\left( \theta _{t},u_{t}\right) ={\tilde{\pi }}_{t}\left( \theta _{t}\right) \psi _{t}\left( u_{t}{\mid }\theta _{t}\right) , \end{aligned}$$

implying $\varphi _{t}$ and $\pi _{t}$ have the same normalising constant $Z_t$. The dimension of $\varTheta _{t}$ can change with *t*, but the dimension of $E_{t}$ must be constant in *t*. We introduce a transformation $G_{t\rightarrow t+1}:\varTheta _{t}\times U_{t}\rightarrow \varTheta _{t+1}\times U_{t+1}$ and define

$$\begin{aligned} \vartheta _{t\rightarrow t+1}=\left( \theta _{t\rightarrow t+1}\left( \vartheta _{t}\right) ,u_{t\rightarrow t+1}\left( \vartheta _{t}\right) \right) :=G_{t\rightarrow t+1}\left( \vartheta _{t}\right) . \end{aligned}$$

In many cases, we will choose $G_{t\rightarrow t+1}$ to be bijective. In this case, we denote its inverse by $G_{t+1\rightarrow t}=G_{t\rightarrow t+1}^{-1}$, with

$$\begin{aligned} \vartheta _{t+1\rightarrow t}&=\left( \theta _{t+1\rightarrow t}\left( \vartheta _{t+1}\right) ,u_{t+1\rightarrow t}\left( \vartheta _{t+1}\right) \right) \\&:=G_{t+1\rightarrow t}\left( \vartheta _{t+1}\right) . \end{aligned}$$

Let the distribution of the transformed random variable $\vartheta _{t\rightarrow t+1}$ be $\varphi _{t\rightarrow t+1}$, i.e., $\varphi _{t\rightarrow t+1}={\mathcal {L}}\left( \vartheta _{t\rightarrow t+1}\right) ={\mathcal {L}}\left( G_{t\rightarrow t+1}\left( \vartheta _{t}\right) \right)$ where ${\mathcal {L}}\left( X\right)$ denotes the law of a random variable *X*, and let the distribution of $\vartheta _{t+1\rightarrow t}$ be $\varphi _{t+1\rightarrow t}$. These distributions may be derived using standard results about the distributions of transforms of random variables: e.g., where the $E_{t}$ are continuous spaces and where $G_{t\rightarrow t+1}$ is a diffeomorphism, having Jacobian determinant $J_{t\rightarrow t+1}$ , with inverse $G_{t+1\rightarrow t}$ having Jacobian determinant $J_{t+1\rightarrow t}$. In this case we have

$$\begin{aligned}&{\tilde{\varphi }}_{t\rightarrow t+1}\left( \vartheta _{t\rightarrow t+1}\right) = {\tilde{\pi }}_{t}\left( \theta _{t+1\rightarrow t}\left( \vartheta _{t\rightarrow t+1}\right) \right) \\&\quad \times \psi _{t}\left( u_{t+1\rightarrow t}\left( \vartheta _{t\rightarrow t+1}\right) {\mid }\theta _{t+1\rightarrow t}\left( \vartheta _{t\rightarrow t+1}\right) \right) \left| J_{t+1\rightarrow t}\right| ,\\&{\tilde{\varphi }}_{t+1\rightarrow t}\left( \vartheta _{t+1\rightarrow t}\right) = {\tilde{\pi }}_{t+1}\left( \theta _{t\rightarrow t+1}\left( \vartheta _{t+1\rightarrow t}\right) \right) \\&\quad \times \psi _{t+1}\left( u_{t\rightarrow t+1}\left( \vartheta _{t+1\rightarrow t}\right) {\mid }\theta _{t\rightarrow t+1}\left( \vartheta _{t+1\rightarrow t}\right) \right) \left| J_{t\rightarrow t+1}\right| . \end{aligned}$$

We may then use an SMC sampler on the sequence of targets $\varphi _{t}$, with the following steps at its $(t+1)$th iteration.

1. **Transform** For the *p*th particle, apply$\vartheta _{t\rightarrow t+1}^{(p)}=G_{t\rightarrow t+1}\left( \vartheta _{t}^{(p)}\right)$.
2. **Reweight and resample** Calculate the updated (unnormalised) weight ${\tilde{w}}_{t+1}^{(p)}$
  9 $$\begin{aligned} {\tilde{w}}_{t+1}^{(p)}= & {} w_{t}^{(p)}\frac{{\tilde{\varphi }}_{t+1}\left( \vartheta _{t\rightarrow t+1}^{(p)}\right) }{{\tilde{\varphi }}_{t\rightarrow t+1}\left( \vartheta _{t\rightarrow t+1}^{(p)}\right) }. \end{aligned}$$
  Where $G_{t\rightarrow t+1}$ is a diffeomorphism we have
  10 $$\begin{aligned} {\tilde{w}}_{t+1}^{(p)}=w_{t}^{(p)}\frac{{\tilde{\pi }}_{t+1}\left( \theta _{t\rightarrow t+1}^{(p)}\right) \psi _{t+1}\left( u_{t\rightarrow t+1}^{(p)}{\mid }\theta _{t\rightarrow t+1}^{(p)}\right) }{{\tilde{\pi }}_{t}\left( \theta _{t}^{(p)}\right) \psi _{t}\left( u_{t}^{(p)}{\mid }\theta _{t}^{(p)}\right) \left| J_{t+1\rightarrow t}\right| }.\nonumber \\ \end{aligned}$$
  It is possible, depending on the transformation used, that this weight update involves none of the dimensions above $\max \left\{ \dim \left( \theta _{t}\right) ,\dim \left( \theta _{t+1}\right) \right\}$ as happened in (4). Then resample if the ESS falls below some threshold, as described previously.
3. **Move** For each *p*, let $\vartheta _{t+1}^{(p)}$ be the result of an MCMC move with target $\varphi _{t+1}$, starting from $\vartheta _{t\rightarrow t+1}^{(p)}$. We need not simulate *u* variables that are not used at the next iteration.

To illustrate the additional flexibility this framework allows, over and above the sampler described in Sect. 2.1.2, we consider the Gaussian mixture example in Sect. 2.2. The sampler from 2.1.2 provides an alternative to RJMCMC in which a set of particles is used to sample from each model in turn, using the particles from model *t*, together with new dimensions simulated using a birth move, to explore model $t+1$. The sampler in this section allows us to use a similar idea using more sophisticated proposals, such as split moves. The efficiency of the sampler depends on the choice of $\psi _{t}$ and $G_{t\rightarrow t+1}$. As previously, a good choice for these quantities should result in a small distance between $\varphi _{t\rightarrow t+1}$ and $\varphi _{t+1}$, whilst ensuring that $\varphi _{t\rightarrow t+1}$ has heavier tails than $\varphi _{t+1}$. As in the design of RJMCMC algorithms, usually these choices will be made using application-specific insight.

### Design of SMC samplers

#### Using intermediate distributions

The Monte Carlo variance of an SMC sampler depends on the distance between successive target distributions; thus, a well-designed sampler will use a sequence of distributions in which the distance between successive distributions is small. We ensure this by introducing intermediate distributions in between successive targets (Neal 2001): in between targets $\varphi _{t}$ and $\varphi _{t+1}$ we use $K-1$ intermediate distributions, the *k*th being $\varphi _{t,k}$, so that $\varphi _{t,0}=\varphi _{t}$ and $\varphi _{t,K}=\varphi _{t+1}$ and therefore $\varphi _{t,K}=\varphi _{t+1,0}$. We use *geometric annealing*, i.e.,

11 $$\begin{aligned}&{\tilde{\varphi }}_{t\rightarrow t+1,k}\left( \vartheta _{t\rightarrow t+1,k}\right) \nonumber \\&\quad = \left[ {\tilde{\varphi }}_{t+1}\left( \vartheta _{t\rightarrow t+1,k}\right) \right] ^{\gamma _{k}}\left[ {\tilde{\varphi }}_{t\rightarrow t+1}\left( \vartheta _{t\rightarrow t+1,k}\right) \right] ^{1-\gamma _{k}}, \end{aligned}$$

where $0=\gamma _{0}<...<\gamma _{K}=1$. This idea results in only small alterations to the TSMC presented above. We now use a sequence of targets $\varphi _{t,k}$, incrementing the *t* index when $k=K$, then setting $k=0$ and finally using a transform move $\vartheta _{t\rightarrow t+1,0}^{(p)}=G_{t\rightarrow t+1}\left( \vartheta _{t,K}^{(p)}\right)$ for each $p\in \left\{ 1,\dots ,P \right\}$. The weight update becomes

12 $$\begin{aligned} {\tilde{w}}_{t,k+1}^{(p)}= & {} w_{t,k}^{(p)}\frac{{\tilde{\varphi }}_{t\rightarrow t+1,k+1}\left( \vartheta _{t\rightarrow t+1,k}^{(p)}\right) }{{\tilde{\varphi }}_{t\rightarrow t+1,k}\left( \vartheta _{t\rightarrow t+1,k}^{(p)}\right) }, \end{aligned}$$

and the MCMC moves now have target $\varphi _{t\rightarrow t+1,k+1}$, starting from $\vartheta _{t\rightarrow t+1,k}^{(p)}$ and storing the result in $\vartheta _{t\rightarrow t+1,k+1}^{(p)}$. The use of intermediate distributions makes this version of TSMC more robust than the previous one; the MCMC moves used at the intermediate distributions provide a means for the algorithm to recover if the initial transformation is not enough to ensure that $\varphi _{t\rightarrow t+1}$ is similar to $\varphi _{t+1}$.

#### Adaptive SMC

Section 2.4.1 describes the use of intermediate distributions with the aim of ensuring that the distance between neighbouring targets is not too great, but this aim cannot be achieved without also considering where to place these intermediate distributions. In this paper, we follow the adaptive strategy used in Jasra et al. (2011) and Del Moral et al. (2012) and refined in Zhou et al. (2015) in the case where resampling is not performed at every iteration. At iteration *t*, $\left( k+1\right)$ this approach uses the conditional ESS (CESS)

13 $$\begin{aligned} \text {CESS}_{t,k+1}=\frac{P\left( \sum _{p=1}^{P}w_{t,k}^{(p)}\omega ^{(p)}\right) ^{2}}{\sum _{p=1}^{P}w_{t,k}^{(p)}\left( \omega ^{(p)}\right) ^{2}}, \end{aligned}$$

to monitor the discrepancy between neighbouring distributions, where $\omega ^{(p)}$ is the incremental weight given by the ratio multiplying $w_{t,k}^{(p)}$ in (12). Before the reweighting step is performed, the next intermediate distribution is chosen to be the distribution under which the CESS is found to be $\beta P$, for some $0<\beta <1$. In the case of the geometric annealing scheme, this corresponds to a particular choice for $\gamma _{k}$ for computing (11). As commented previously, we may also adapt the MCMC kernels used for the move step, based on the current particle set. For the two examples presented later, we have considered adaptive and non-adaptive strategies in the MCMC kernels. We refer the interested reader to the supplementary material for the specific details. Algorithm 1 presents a generic version of TSMC using adaptive resampling and number of intermediate distributions.

#### Auxiliary variables in proposals

For the Gaussian mixture example, for two or more components, when using a split move we must choose the component that is to be split. We may think of the choice of splitting different components as offering multiple “routes” through a space of distributions, with the same start and end points. Another alternative route would be given by using a birth move rather than a split move. In this section, we generalise TSMC to allow multiple routes. We restrict our attention to the case where the choice of multiple routes is possible at the beginning of a transition from $\varphi _{t}$ to $\varphi _{t+1}$, when $k=0$ (more general schemes are possible). A route corresponds to a particular choice for the transformation $G_{t\rightarrow t+1}$; thus, we consider a set of $M_{t}$ possible transformations indexed by the discrete random variable $l_{t}$, using the notation $G_{t\rightarrow t+1}^{\left( l_{t}\right) }$ (also using this superscript on distributions that depend on this choice of *G*). We now augment the target distribution with variables $l_{0},...,l_{T-1}$ and, for each *t* alter the distribution $\psi _{t}$ such that it becomes a joint distribution on $u_{t}$ and $l_{t}$. Our sampler will draw the *l* variables at the point at which they are introduced, so that different particles use different routes, but will not perform any MCMC moves on the variable after it is introduced. This leads to the sampler being degenerate in most of the *l* variables, but this doesn’t affect the desired target distribution.

A revised form of TSMC is then, when $k=0$, to first simulate routes $l_{t}^{(p)}\sim \rho _{t}$ for each particle, then to use a different transform $\vartheta _{t\rightarrow t+1,0}^{(p)}=G_{t\rightarrow t+1}^{\left( l_{t}^{(p)}\right) }\left( \vartheta _{t,K}^{(p)}\right)$ dependent on the route variable. The weight update is then given by

14 $$\begin{aligned} {\tilde{w}}_{t+1}^{(p)}=w_{t}^{(p)}\frac{{\tilde{\pi }}_{t+1}\left( \theta _{t\rightarrow t+1}^{(p)}\right) \psi _{t+1}\left( u_{t\rightarrow t+1}^{(p)},l_{t}^{(p)}{\mid }\theta _{t\rightarrow t+1}^{(p)}\right) }{{\tilde{\pi }}_{t}\left( \theta _{t}^{(p)}\right) \psi _{t}\left( u_{t}^{(p)},l_{t}^{(p)}{\mid }\theta _{t}^{(p)}\right) \left| J_{t+1\rightarrow t}^{\left( l_{t}^{(p)}\right) }\right| },\nonumber \\ \end{aligned}$$

where for simplicity we have omitted the dependence of $u_{t}^{(p)}$, $u_{t\rightarrow t+1}^{(p)}$ and $\theta _{t\rightarrow t+1}^{(p)}$ on $l_{t}^{(p)}$. This weight update is very similar to one found in Del Moral et al. (2006), for the case where a discrete auxiliary variable is used to index a choice of MCMC kernels used in the move step. Analogous to Del Moral et al. (2006), the variance of (14) is always greater than or equal to that of (10); we present an example in Sect. 3 where this additional variance can result in large errors in marginal likelihood estimates). Alternatively one can employ the Rao–Blackwellisation procedure found in population Monte Carlo (Douc et al. 2007) and marginalise the proposal over the auxiliary variable $l_{t}$. This results in a weight update of

15 $$\begin{aligned} {\tilde{w}}_{t+1}^{(p)}=w_{t}^{(p)}\frac{\pi _{t+1}\left( \theta _{t\rightarrow t+1}^{(p)}\right) \psi _{t+1}\left( u_{t\rightarrow t+1}^{(p)}{\mid }\theta _{t\rightarrow t+1}^{(p)}\right) }{\sum _{m=1}^{M_{t}}\pi _{t}\left( \theta _{t}^{(p)}\right) \psi _{t}\left( u_{t}^{(p)},m{\mid }\theta _{t}^{(p)}\right) \left| J_{t+1\rightarrow t}^{\left( m\right) }\right| }.\nonumber \\ \end{aligned}$$

As mentioned in Del Moral et al. (2006), using (15) comes with extra computational cost, which could be prohibitively large if $M_t$ is large.
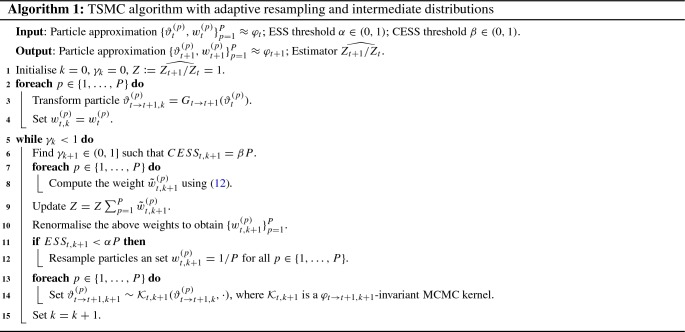


### Discussion

One of the most obvious applications of TSMC is Bayesian model comparison. SMC samplers are a generalisation of several other techniques, such as IS, AIS and the “stepping stone” algorithm from Xie et al. (2011) (which is essentially equivalent to AIS where more than one MCMC move is used per target distribution); thus, we expect a well-designed SMC to outperform these techniques in most cases. Zhou et al. (2015) reviews existing techniques that use SMC for model comparison and concludes that “the SMC2 algorithm (moving from prior to posterior) with adaptive strategies is the most promising among the SMC strategies.” In Sect. 3, we provide a detailed comparison of TSMC with SMC2 and find that TSMC can have significant advantages.

Section 2.2.2 compared TSMC with RJMCMC, noting that RJMCMC explores the model space by using a high variance estimator of a Bayes factor at each MCMC iteration, whereas TSMC is designed to construct a single lower variance estimator of each Bayes factor. The high variance estimators within RJMCMC are the cause of its most well-known drawback: that the acceptance rate of trans-dimensional moves can be very small. The design of TSMC, in which each model is visited in turn, completely avoids this issue. One might envisage that despite avoiding poor mixing, TSMC might instead yield high variance Bayes factor estimators for challenging problems. However, TSMC has the advantage that that adaptive methods may be used in order to reduce the possibility that the estimators have high variance by, for example, automatically using more intermediate distributions. The possibility to adaptively choose intermediate distributions also provides an advantage over the approach of Karagiannis and Andrieu (2013), where a sequence of intermediate distributions for estimating each Bayes factor must be specified in advance.

Since, by construction, TSMC is a particular instance of SMC as described in Del Moral et al. (2006), all of the theoretical properties of a standard SMC algorithm apply. Of particular interest are the properties of the method as the dimension of the parameter spaces grows. TSMC is constructed on a sequence of extended spaces $E_t$, each of which has dimension $d_{T}$, thus in the worst case, the results for an SMC sampler on a space of dimension $d_{T}$ apply. In this respect, the authors in Beskos et al. (2014) have analysed the stability of SMC samplers as the dimension of the state space increases when the number of particles *P* is fixed. Their work provides justification, to some extent, for the use of intermediate distributions $\left( \varphi _{t,k}\right) _{k=1}^{K}$. Under fairly strong assumptions, it has been shown that when the number of intermediate distributions $K={\mathcal {O}}\left( d_{T}\right)$, and as $d_{T}\rightarrow \infty$, the effective sample size $\text{ ESS }_{t+1}^{P}$ is stable in the sense that it converges to a non-trivial random variable taking values in $\left( 1,P\right)$. The total computational cost for bridging $\varphi _{t}$ and $\varphi _{t+1}$, assuming a product form of $d_{T}$ components, is ${\mathcal {O}}\left( Pd_{T}^{2}\right)$. However, in practice, due to the cancellation of “fill in” variables, and using sensible transformations between consecutive distributions, one could expect a much lower effective dimension of the problem; an example of this situation is presented in the next section. Some theoretical properties of the method are explored further in the Supplementary Information.

## Bayesian model comparison for mixtures of Gaussians

In this section, we examine the use of TSMC on the mixture of Gaussians application in Sect. 2.2: i.e., we wish to perform Bayesian inference of the number of components *t*, and their parameters $\theta _{t}$, from data *y*. For simplicity, we study the “without completion” model, where component labels for each measurement are not included in the model. In the next sections, we outline the design of the algorithms used, then in Sect. 3.2 we describe the results of using these approaches on previously studied data, highlighting features of the approach. Further results are given in the Supplementary Information.

### Description of algorithms

Let *t* be the unknown number of mixture components, and $$\left( \mu _{1:t},\tau _{\text {1:t}},\nu _{1:t}\right) $$ (means, precisions and weights respectively) be the parameters of the *t* components. Our likelihood is the same as in Eq. (5); we use priors $$\tau \sim \text {Gamma}\left( 2,2S^{2}/100\right) ,$$$$\nu _{1:t}\sim \text {Dir}\left( 1,...,1\right) $$ for the precisions and weights, respectively, and for the means we choose an unconstrained prior of $$\mu \sim {\mathcal {N}}\left( m,S^{2}\right) $$, where *m* is the mean and *S* is the range of the observed data. We impose an ordering constraint on the means, as described in Jasra et al. (2005), which simplifies the problem by eliminating many posterior modes with the added benefit of improving the interpretability of our results. For simplicity, we have also not included the commonly used “random beta” hierarchical prior structure on $$\tau $$ (Richardson and Green 1997), which from a statistical perspective is suboptimal but which simplifies our presentation of the behaviour of TSMC.

We use different variants of TSMC (as described in Sect. 2.3), using a sequence of distributions $\left( \varphi _{t}\right) _{t=1}^{T}$ where $\varphi _{t}\left( \vartheta _{t}\right) =\pi _{t}\left( \theta _{t}\right) \psi _{t}\left( u_{t}\right)$. $\pi _{t}$ is here the posterior on *t* components given by Eq. (6), and $\psi _{t}$ is different depending on the transformation that is chosen. We use intermediate distributions (as described in Sect. 2.4.1), using geometric annealing, in all of our algorithms, making use of the adaptive method from Sect. 2.4.2 to choose how to place these distributions. The results in this section focus particularly on illustrating the advantages afforded by making an intelligent choice of the transformation in TSMC. Full details of the transformations, weight updates and MCMC moves are given in the Supplementary Information. In summary, we use the birth and split moves referred to in Sect. 2.2, together with a move that orders the components. For both moves, we present results using the weight updates in Eqs. (14) (referred to henceforth as the conditional approach) and (15) (referred to as the marginal approach).

### Results

**Fig. 1:**
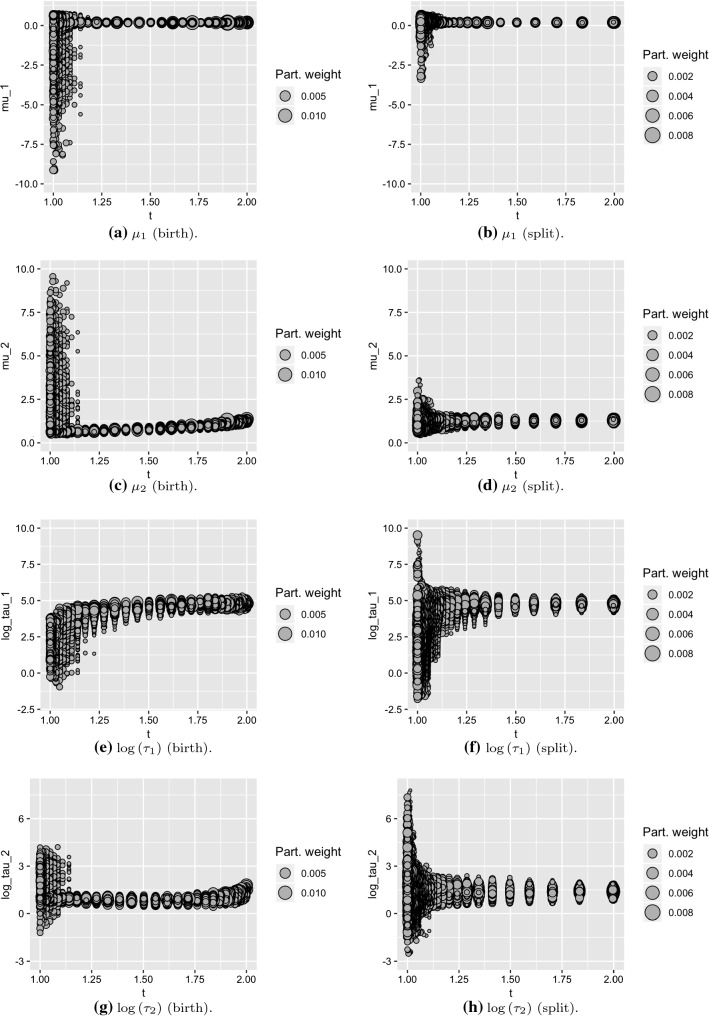
The evolution of particles from model 1 to model 2 for the birth and split moves on the enzyme data

We ran SMC2 and the TSMC approaches on the enzyme data from Richardson and Green (1997). We ran the algorithms 50 times, up to a maximum of $T=8$ components, with $P=500$ particles. We used an adaptive sequence of intermediate distributions, choosing the next intermediate distribution to be the one that yields a CESS (Eq. 13) of $\beta P$, where $\beta =0.99$. We resampled using stratified resampling when the ESS falls below $\alpha P$, where $\alpha =0.5$. Figure 1 compares the birth and split TSMC algorithms when moving from one to two components. We observe that the split transformation has the effect of moving the parameters to initial values that are more appropriate for exploring the posterior on two components. For this dataset, the birth move is a poor choice for the existing parameters in the model: Fig. 1e shows that no particles drawn from the proposal (i.e., the posterior for the single component model) overlap with the posterior for the first component in the two component model. Despite the poor proposal, the intermediate distributions (of which there are many more than used for the split move) enable a good representation of the posterior distribution, although below we see that the poor proposal results in very poor estimates of the marginal likelihood.

**Fig. 2:**
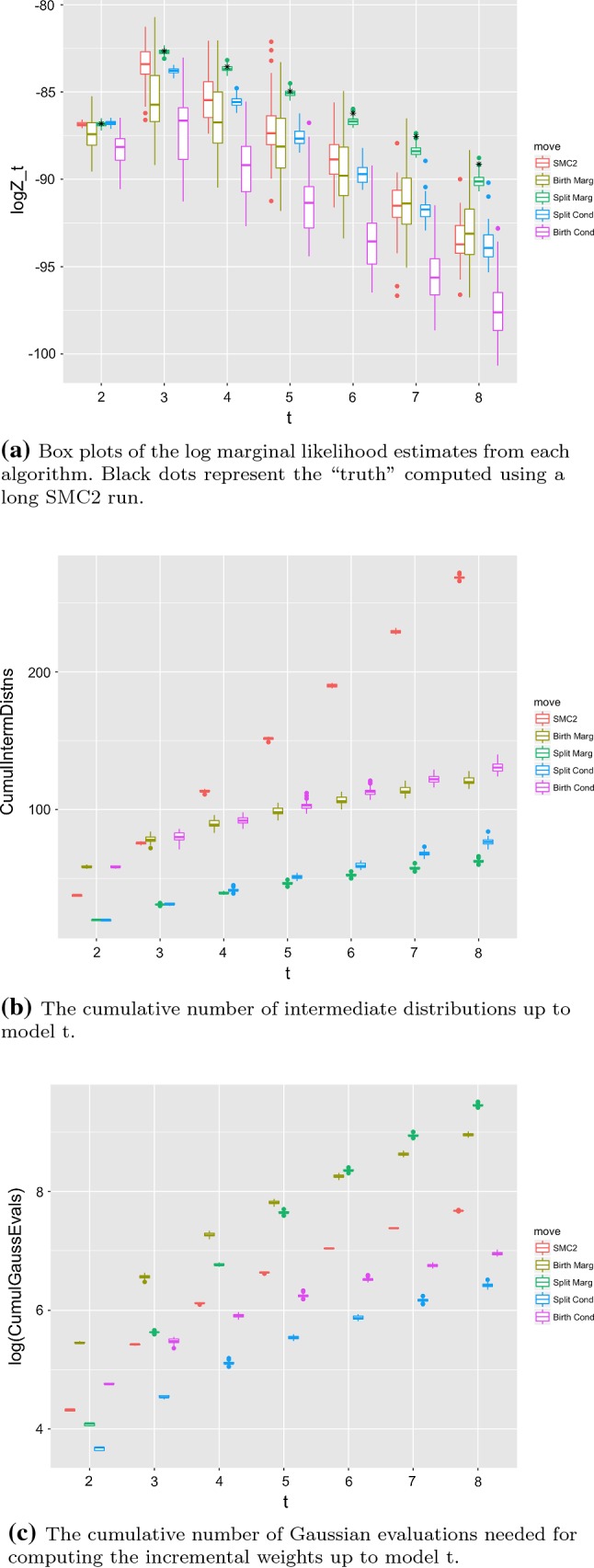
The relative performance of the different SMC schemes on the mixture example

Figure 2a shows log marginal likelihood estimates from the different approaches (note that a poor quality SMC usually results in an underestimate of the log marginal likelihood), and the cumulative number of intermediate distributions used in estimating all of the marginal likelihoods up to model *t* for each $t\in \{ 1, \dots ,T \}$. We observe that the performance of SMC2 degrades as the dimension increases due to the increasing distance of the prior from the posterior: we see that the adaptive scheme using the CESS results in the number of intermediate distributions across all dimensions being approximately constant which, as suggested by Beskos et al. (2014) is insufficient to control the variance as the dimension grows. As discussed above, both birth TSMC methods yield inaccurate Bayes’ factor estimates, with split TSMC exhibiting substantially better performance. However, we see that neither conditional approach yields very accurate results when using the weight update given in Eq. (14); instead the marginalised weight update is required to provide good estimates. The marginal version of split TSMC significantly outperforms the other approaches, although we note that this is achieved at a higher computational cost due to the sum in the denominator of the weight updates, this can be observed in Fig. 2c which shows the cumulative number of Gaussian evaluations for computing the weights in each case. For all TSMC approaches, we see that the number of intermediate distributions (Fig. 2b) decreases as we increase dimension. This result can be attributed to the relatively small change that results from only adding a single component to the model at a time in TSMC. If the method has a good representation of the target at model *t* and there is minimal change in the posterior on the existing *t* components when moving to model $t+1$, then the SMC is effectively only exploring the posterior on the additional component and thus has higher ESS.

In the Supplementary Information, we provide similar results for two other datasets, stressing that sensible transformations and efficient MCMC moves are essential for obtaining good estimates of the normalising constants. Interestingly, and in contrast to the enzyme data presented above, for one of these other datasets neither the split nor the birth moves outperformed SMC2; this is due to the specific distribution of the observations in such dataset.

## Sequential Bayesian inference under the coalescent

### Introduction

In this section, we describe the use of TSMC for online inference under the coalescent model in population genetics (Kingman 1982); we consider the case in which we wish to infer the *clonal ancestry* (or *ancestral tree*) of a bacterial population from DNA sequence data. Current approaches in this area use MCMC (Drummond and Rambaut 2007), which is a limitation in situations where DNA sequence data does not arrive as a batch, such as may happen when studying the spread of an infectious disease as the outbreak is progressing (Didelot et al. 2014). We instead introduce an SMC approach to online inference, inferring posterior distribution as sequences become available (this approach is similar to that of Dinh et al. (2018) which was devised simultaneously to ours). We further envisage that TSMC will be useful in cases in which data is available as a single batch, through exploiting the well-known property that a tree estimated from $t+1$ sequences is usually similar to a tree estimated from *t* sequences. Exploring the space of trees for a large number of sequences appears challenging due to the large number of possible trees: through adding leaves one by one the SMC approach follows a path through tree space in which transitions from distribution $\pi _{t}$ to $\pi _{t+1}$ are not challenging. Further, our approach yields more stable estimates of the marginal likelihood of models than current approaches used routinely in population genetics, such as the infinite variance harmonic mean estimator (Drummond and Rambaut 2007) and the stepping stone algorithm (Drummond and Rambaut 2007; Xie et al. 2011).

#### Previous work

The idea of updating a tree by adding leaves dates back to at least Felsenstein (1981), in which he describes, for maximum likelihood estimation, that an effective search strategy in tree space is to add species one by one. More recent work also makes use of the idea of adding sequences one at a time: ARGWeaver (Rasmussen et al. 2014) uses this approach to initialise MCMC on (in this case, a space of graphs), $t+1$ sequences using the output of MCMC on *t* sequences, and TreeMix (Pickrell and Pritchard 2012) uses a similar idea in a greedy algorithm. In work conducted simultaneously to our own, Dinh et al. (2018) also propose a sequential Monte Carlo approach to inferring phylogenies in which the sequence of distributions is given by introducing sequences one by one. However, their approach: uses different proposal distributions for new sequences; does not infer the mutation rate simultaneously with the tree; does not exploit intermediate distributions to reduce the variance; and does not use adaptive MCMC moves. Further investigation of their approach can be found in Fourment et al. (2018), where different guided proposal distributions are explored but that still presents the aforementioned limitations.

#### Data and model

We consider the analysis of *T* aligned genome sequences $y=y_{1:T}$, each of length *N*. Sites that differ across sequences are known as single nucleotide polymorphisms (SNPs). The data (which is freely available from http://pubmlst.org/saureus/) used in our examples consists of seven “multi-locus sequence type” (MLST) genes of 25 *Staphylococcus aureus* sequences, which have been chosen to provide a sample representing the worldwide diversity of this species (Everitt et al. 2014). We make the assumption that the population has had a constant size over time, that it evolves clonally and that SNPs are the result of mutation. Our task is to infer the clonal ancestry of the individuals in the study, i.e., the tree describing how the individuals in the sample evolved from their common ancestors, and [additional to Dinh et al. (2018)] the rate of mutation in the population. We describe a TSMC algorithm for addressing this problem in Sect. 4.2, before presenting results in Sect. 4.3. In the remainder of this section, we introduce a little notation.

Let ${\mathcal {T}}_{t}$ represent the clonal ancestry of *t* individuals and let $\theta /2$ be the expected number of mutations in a generation. We are interested in the sequence of distributions

$$\begin{aligned} \pi _{t}\left( {\mathcal {T}}_{t},\theta {\mid } y_{1:t}\right) \propto f\left( y_{1:t}{\mid }{\mathcal {T}}_{t},\theta \right) p\left( {\mathcal {T}}_{t}\right) p\left( \theta \right) \end{aligned}$$

for $t=1:T$. We here we use the coalescent prior (Kingman 1982) $p\left( {\mathcal {T}}_{t}\right)$ for the ancestral tree, the Jukes-Cantor substitution model (Jukes and Cantor 1969) for $f\left( y_{1:t}{\mid }{\mathcal {T}}_{t},\theta \right)$ and choose $p\left( \theta \right)$ to be a gamma distribution with shape 1 and rate 5 (that has its mass on biologically plausible values of $\theta$). Let $l_{t}^{(a)}$ denote the length of time for which *a* branches exist in the tree, for $2\le a\le t$. The heights of the coalescent events are given by $h^{(a)}=\sum _{\iota =a}^{t}l_{t}^{(\iota )}$, with $h_{t}^{(a)}$ being the $\left( t-a+1\right)$th coalesence time when indexing from the leaves of the tree. We let ${\mathcal {T}}_{t}$ be a random vector $\left( {\mathcal {B}}_{t},h_{t}^{(2)},...,h_{t}^{(t)}\right)$ where ${\mathcal {B}}_{t}$ is itself a vector of discrete variables representing the branching order. When we refer to a lineage of a leaf node, this refers to the sequence of branches from this leaf node to the root of the tree.

### TSMC for the coalescent

In this section, we describe an approach to adding a new leaf to an existing tree, using a transformation as in Sect. 2.3. The basic idea is to first propose a lineage to add the new branch to (from distribution $\chi _{t}^{(g)}$), followed by a height $h_{\text {t}}^{(\text {new})}$ conditional on this lineage (from distribution $\chi _{t}^{(h)}$) at which the branch connected to the new leaf will join the tree. The resultant weight update is

16 $$\begin{aligned}&{\tilde{w}}_{t+1}=w_{t} \frac{\pi _{t+1}\left( {\mathcal {T}}_{t+1},\theta {\mid } y_{1:t+1}\right) }{\pi _{t}\left( {\mathcal {T}}_{t},\theta {\mid } y_{1:t}\right) } \nonumber \\&\quad \Bigg / \Bigg (\sum _{s\in \varLambda } \Big [ \chi _{t}^{(g)}\left( g_{t}=s{\mid }\theta _{t},{\mathcal {T}}_{t},y_{1:t+1}\right) \nonumber \\&\qquad \times \chi _{t}^{(h)} \left( h_{t}^{(\text {new})}{\mid } g_{t}=s,\theta _{t},{\mathcal {T}}_{t},y_{1:t+1} \right) \Big ] \Bigg ) \end{aligned}$$

where $\varLambda$ is the set that contains the leaves of the lineages that if proposed, could have resulted in the new branch (under the inverse image of the transformation). Note the relationship with Eq. (15): we achieve a lower variance through summing over the possible lineages rather than using an SMC over the joint space that includes the lineage variable.

To choose the lineage, we make use of an approximation to the probability that the new sequence is $M_{s}$ mutations from each of the existing leaves, via approximating the pairwise likelihood of the new sequence and each existing leaf. Following Stephens and Donnelly (2000) (see also Li and Stephens (2003)), we set the probability of choosing the lineage with leaf *s* using

17 $$\begin{aligned} \chi _{t}^{(g)}\left( s{\mid }\theta _{t},y_{1:t+1}\right) \propto \left( \frac{N\theta _{t}}{t+N\theta _{t}}\right) ^{M_{s}}. \end{aligned}$$

For $\chi _{t}^{(h)}$, we propose to approximate the pairwise likelihood $f_{t+1,s}\left( y_{s},y_{t+1}{\mid }\theta ,h_{t}^{(\text {new})},g_{t}=s\right)$, where $y_{s}$ is the sequence at the leaf of the chosen lineage. Since only two sequences are involved in this likelihood, it is likely to have heavier tails than the posterior. We use a Laplace approximation on a transformed space, following Reis and Yang (2011): further details are given in the Supplementary Information, Sect. 3.2.

### Results

We used $P=250$ particles, with an adaptive sequence of intermediate distributions, choosing the next intermediate distribution to be the one the yields a CESS (Eq. 13) of $\beta P$, where $\beta =0.95$. Resampling is performed whenever the ESS falls below $\alpha P$, where $\alpha =0.5$. At each iteration we used the current population of particles to tune the proposal variances, as detailed in the Supplementary Information, section 3.3.

We used six different configurations of our approach, for two different orderings of the 25 sequences. The two orderings were chosen as follows: the “nearest”/“furthest” ordering was chosen by starting with the two sequences with the smallest/largest pairwise SNP difference, then add sequences in the order of minimum/maximum SNP difference to an existing sequence. The six configurations of the methods were: the default configuration; using no tree topology changing MCMC moves; taking $\chi _{t}^{(h)}$ to be an $\text {Exp}(1)$ distribution (less concentrated than the Laplace-based proposal); raising Eq. (17) to the power 0 to give a uniform lineage proposal; raising Eq. (17) $\chi _{t}^{(g)}$ to the power 2; and raising Eq. (17) $\chi _{t}^{(g)}$ to the power 4. These latter two approaches use a lineage proposal where the probability is more concentrated on a smaller number of lineages.

**Fig. 3:**
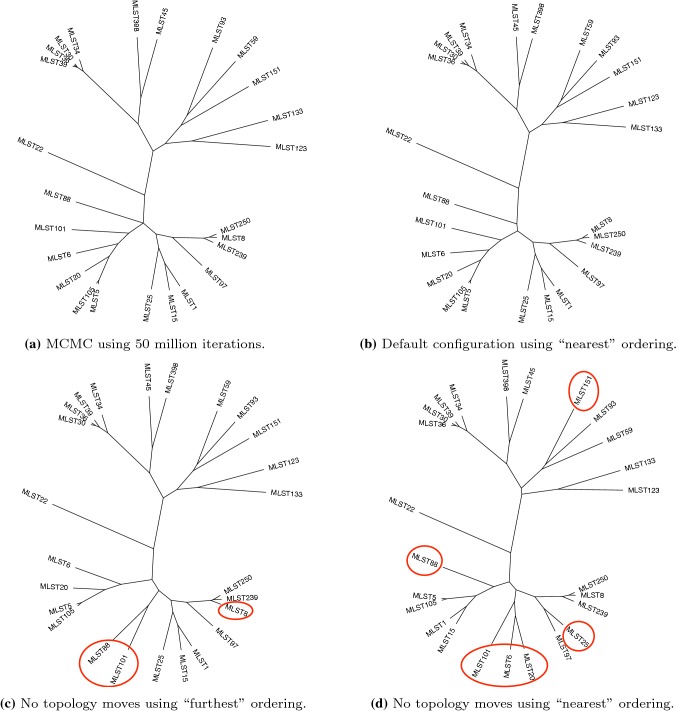
Majority-rule consensus trees found by MCMC and the default configuration of TSMC (top), and different configurations of TSMC (bottom) with differences to the result obtained by the default configuration highlighted

**Table 1:** Log marginal likelihood estimates and total number of distributions for TSMC applied to the coalescent (5 s.f.), for the “Furthest” (first line) and “Nearest” (second line) orderings

Default	No top. moves	$\chi _{t}^{(h)}=\text {Exp}(1)$	$\left( \chi _{t}^{(h)}\right) ^{0}$	$\left( \chi _{t}^{(h)}\right) ^{2}$	$\left( \chi _{t}^{(h)}\right) ^{4}$
$-$ 6333.9/267	$-$ 6338.8/257	$-$ 6335.1/408	$-$ 6336.9/330	$-$ 6333.1/247	$-$ 6334.3/238
$-$ 6335.8/323	$-$ 6354.6/293	$-$ 6337.8/501	$-$ 6341.0/384	$-$ 6339.0/300	$-$ 6342.0/255

Figure 3 shows majority-rule consensus trees from an MCMC run and the final TSMC iterations. Figure 3b is generated by the default configuration (for the “furthest” ordering, although results from the “nearest” ordering are nearly identical) and is close to the ground truth in Fig. 3a (as determined by a long MCMC run). Figure 3c, d used no topology changing MCMC moves, thus illustrating the contribution of the SMC proposal in determining the topology. Table 1 shows estimates of the log marginal likelihood from each configuration of the algorithm for both orderings (longer runs of our method suggest the true value is $\approx -\,6333$), along with the total number of intermediate distributions used. Recall that a poorer-quality SMC usually results in an underestimate of the log marginal likelihood, and the number of intermediate distributions offers an indication as to the distance between the target and the proposal where the proposal has heavier tails than the target. We draw the following conclusions:

- As also suggested by Fig. 3, we see that the “furthest” ordering provides consistently better results than the “nearest” ordering. “Furthest” provides an ordering in which new sequences are often added above the root of the current tree, since the existing sequences are all more closely related than the new sequence, whereas “nearest” frequently results in adding a leaf close to the existing leaves of the tree. In the latter strategy, the proposal relating to the new sequence is often good, but adding a new sequence can have a large effect on the posterior of existing variables. We see this by comparing Fig. 3c, d, observing that the “furthest” ordering results in a topology that is close to the truth. The topology from the “nearest” ordering is not as close to the truth, thus is more reliant on topology changing MCMC moves to give an accurate sample from the posterior.
- As expected, using no MCMC topology moves results in very poor estimates, highlighting the important role of MCMC in generating diversity not introduced in the SMC proposals. This poor quality is not accounted for by the adaptive scheme based on the CESS introducing more intermediate distributions, since the CESS is only based on the weights of the particles and cannot account for a lack of diversity.
- Using less directed proposals, on both the lineage and the height, increases the distance between the proposal and target, and results in lower quality estimates.
- Using more directed proposals on the lineage may in some cases slightly improve the method, but appear to make the method less robust to the order in which the individuals are added (so may not be suitable in applications where the order of the individuals cannot be chosen).

A video showing the evolution of the majority-rule consensus tree (and the marginal likelihood estimate) through all iterations of the SMC, using the default configuration, can be found at https://www.youtube.com/watch?v=pSDK9ajm2OY.

## Conclusions

This paper introduces a sequential technique for Bayesian model comparison and parameter estimation, and an approach to online parameter and marginal likelihood estimation for the coalescent, underpinned by the same methodological development: TSMC. We show that whilst TSMC performs inference on a sequence of posterior distributions with increasing dimension, it is a special case of the standard SMC sampler framework of Del Moral et al. (2007). In this section, we outline several points that are not described elsewhere.

One innovation introduced in the paper is the use of transformations within SMC for creating proposal distributions when moving between dimensions. The effectiveness of TSMC is governed by the distance between neighbouring distributions; thus, to design TSMC algorithms suitable for any given application, we require the design of a suitable transformation that minimises the distance between neighbouring distributions. This is essentially the same challenge as is faced in designing effective RJMCMC algorithms, and we may make use of many of the methods devised in the RJMCMC literature (Hastie and Green 2012). The ideal case is to use a transformation such that every distribution $\varphi _{t\rightarrow T}$ becomes identical, in which case one may simulate from $\pi _{T}$ simply by simulating from $\pi _{0}$ then applying the transformation. Approximating such a “transport map” for a sequence of continuous distributions is described in Heng et al. (2015). As discussed in Sect. 1.2, Heng et al. (2015) is one of a number of papers that seeks to automatically construct useful transformations, and we anticipate these techniques being of use in the case of changing dimension that is addressed in this paper. In the RJMCMC literature, Brooks et al. (2003) describe methods for automatically constructing the “fill in” distributions $\psi _{t}$ for a given transformation: the literature on transport maps could be used to automatically construct the transformation in advance of this step.

In Fig. 2 of Sect. 3, we see a characteristic of this approach that will be common to many applications, in that the estimated marginal likelihood rises as the model is improved, then falls as the effect of the model complexity penalisation becomes more influential than improvements to the likelihood. We note that by using estimates of the variance of the marginal likelihood estimate (Lee and Whiteley 2015), we may construct a formal diagnostic that decides to terminate the algorithm at a particular model, on observing that the estimated marginal likelihood declines from an estimated maximum value.

Although the examples in this paper both involve posterior distributions of increasing dimension, we also see a use for our approach in some cases that involve a distributions of decreasing dimension. For example, in population genetics, it is common to perform a large number of different analyses using different overlapping sets of sequences. For this reason, many practitioners would value an inference technique that allows for the removal, as well as the addition, of sequences. Further, many genetics applications now involve the analysis of whole genome sequences. Our approach is applicable in this setting, and for this purpose a BEAST2 package is currently under development.

## Electronic supplementary material

Below is the link to the electronic supplementary material.

Supplementary material 1 (zip 6 KB)

Supplementary material 2 (pdf 1163 KB)
