# Supplementary material for: Sequential Monte Carlo with transformations
Source: Stat Comput. 2019 Nov 17;30(3):663–76. doi: 10.1007/s11222-019-09903-y (PMC7026014; doi:10.1007/s11222-019-09903-y)
Supplement: Supplementary file 2 — Supplementary material 2 (pdf 1163 KB) [file 11222_2019_9903_MOESM2_ESM.pdf]

Supplementary material

29th August 2019

1 Theoretical aspects

We define a sequence of targets $(\varphi_{t\to T})_{t=0}^{T}$ on the same measurable space $(E_T, \mathcal{E}_T)$ as follows. Consider $\vartheta_t \sim \varphi_t(\cdot)$ and define

$$\varphi_{t\to T}(\cdot) \quad := \quad \mathcal{L}\left(G_{t\to T}(\vartheta_t)\right),$$

where $\mathcal{L}(X)$ denotes the law of a random variable X, and where $G_{t\to T}$ is defined recursively for $0 \le t < T$ as follows

$$G_{t\to T} := G_{t+1\to T} \circ G_{t\to t+1},$$

with $G_{T\to T}$ as the identity function. As before, the distributions $\varphi_{t\to T}$, φ_t and π_t all share the same normalising constant Z_t. Hence, the TSMC algorithm described in the main paper can be seen as an SMC sampler for the targets $\{\varphi_{t\to T}\}_t$, propagating particles $\left\{\vartheta_{t\to T}^{(p)}\right\}_{t,p}$ using a sequence of MCMC kernels $\{K_{t\to T} : E_T \times \mathcal{E}_T \to [0,1]\}_t$, where $K_{t\to T}$ admits $\varphi_{t\to T}$ as invariant. This will also be the case for the modified TSMC algorithm with intermediate distributions, however the details are omitted for simplicity.

Therefore, after the $(t+1)$th iteration the target $\varphi_{t+1\to T}$ can be approximated using

$$\hat{\varphi}_{t+1\to T}^{P} \quad = \quad \sum_{p=1}^{P} w_{t+1\to T}^{(p)} \delta_{\vartheta_{t+1\to T}^{(p)}},$$

where, for every $p \in \{1,\ldots,P\}$,

$$w_{t+1\to T}^{(p)} \quad \propto \quad w_{t\to T}^{(p)} \frac{\tilde{\varphi}_{t+1\to T}\left(\vartheta_{t\to T}^{(p)}\right)}{\tilde{\varphi}_{t\to T}\left(\vartheta_{t\to T}^{(p)}\right)}$$

and $w_{0\to T}^{(p)} = 1/P$. Furthermore, notice that $w_{t\to T}^{(p)} = w_t^{(p)}$ for all $0 \le t < T$ and any $p \in \{1,\ldots,P\}$, consequently expectations of the form $\varphi_{t+1}(h)$ (for a function $h : E_t \to \mathbb{R}$) can be approximated using

$$\begin{aligned}\hat{\varphi}_{t+1\to T}^{P}\left(h \circ G_{T\to t+1}\right) \quad &= \quad \sum_{p=1}^{P} w_{t+1\to T}^{(p)} h \circ G_{T\to t+1}\left(\vartheta_{t+1\to T}^{(p)}\right) \\ &= \quad \sum_{p=1}^{P} w_{t+1}^{(p)} h\left(\vartheta_{t+1}^{(p)}\right) = \hat{\varphi}_{t+1}^{P}(h).\end{aligned}$$

The following theorem, the proof of which may be found in Del Moral et al. (2006, Proposition 2), follows from well-known standard SMC convergence results.

Theorem 1.1. *Under weak integrability conditions (see Chopin, 2004, Theorem 1 or Del Moral, 2004, p300-306) and for any bounded $h : E_t \to \mathbb{R}$, as $P \to \infty$*

1. $P^{1/2}\left\{\hat{\varphi}_t^P(h) - \bar{\varphi}_t(h)\right\} \Rightarrow \mathcal{N}\left(\cdot \,\middle|\, 0, \sigma_{IS,t}^2(h)\right)$, if no resampling is performed;

2. $P^{1/2}\left\{\hat{\varphi}_t^P(h) - \bar{\varphi}_t(h)\right\} \Rightarrow \mathcal{N}\left(\cdot \,\middle|\, 0, \sigma_{SMC,t}^2(h)\right)$, when multinomial resampling is performed at every iteration;

where $\sigma^2_{IS,t}(h)$ and $\sigma^2_{SMC,t}(h)$ follow similar expressions to those in Del Moral et al. (2006, Proposition 2).

Remark 1.1. As noted also in Del Moral et al. (2006), under strong mixing assumptions, the variance $\sigma^2_{SMC,t}(h)$ can be uniformly bounded in t whereas $\sigma^2_{IS,t}(h)$ will typically diverge as t increases.

Respecting the normalising constants $\{Z_t\}_{t=1}^T$, they can be approximated using

$$\hat{Z}^P_{t+1} = \prod_{s=1}^{t+1}\sum_{p=1}^{P} w^{(p)}_{s\to T}\frac{\tilde{\varphi}_{s+1\to T}\left(\vartheta^{(p)}_{s\to T}\right)}{\tilde{\varphi}_{s\to T}\left(\vartheta^{(p)}_{s\to T}\right)} = \prod_{s=1}^{t+1}\sum_{p=1}^{P} w^{(p)}_{s}\frac{\tilde{\varphi}_{s+1\to s}\left(\vartheta^{(p)}_{s}\right)}{\tilde{\varphi}_{s}\left(\vartheta^{(p)}_{s}\right)},$$

and standard results show that these estimates are unbiased (see e.g. Del Moral, 2004, proposition 7.4.1), with relative variance increasing at most linearly in t (Cérou et al., 2011, Theorem 5.1). Such results are summarised in the following theorem.

Theorem 1.2. *For fixed E_T, and when resampling is not done adaptively, the estimates $\left\{\hat{Z}^P_t\right\}_t$ satisfy*

$$\mathbb{E}\left[\hat{Z}^P_t\right] = Z_t.$$

Furthermore, under strong mixing assumptions there exists a constant $C_T(t)$, which is linear in t, such that

$$\mathbb{V}\left[\frac{\hat{Z}^P_t}{Z_t}\right] \le \frac{C_T(t)}{P}.$$

However, as T increases the dimension of E_T (denoted hereafter by d_T) may increase and we will usually require an exponential growth in the number of particles P in order to obtain meaningful results, see e.g. Bickel et al. (2008). For instance, without the resampling step the ESS at time $t+1$ is closely related to the following quantity (see e.g. Agapiou et al., 2017)

$$\rho_{t+1}(d_T) := \mathbb{E}\left[\left(\prod_{s=1}^{t+1}\frac{\varphi_{s\to T}(\vartheta_{s-1\to T})}{\varphi_{s-1\to T}(\vartheta_{s-1\to T})}\right)^2\right],$$

which serves as a measure of the dissimilarity between proposals and targets, and that quite often increases exponentially in d_T. This quantity provides information about the limiting proportion of effective number of particles since

$$\lim_{P\to\infty}\left(\frac{\mathrm{ESS}^P_{t+1}}{P}\right)^{-1} = \lim_{P\to\infty} P\sum_{p=1}^{P}\left(w^{(p)}_{t+1\to T}\right)^2 = \lim_{P\to\infty}\frac{\frac{1}{P}\sum_{p=1}^{P}\left(w^{(p)}_0\prod_{s=1}^{t+1}\frac{\tilde{\varphi}_{s\to T}\left(\vartheta^{(p)}_{s-1\to T}\right)}{\tilde{\varphi}_{s-1\to T}\left(\vartheta^{(p)}_{s-1\to T}\right)}\right)^2}{\left(\frac{1}{P}\sum_{p=1}^{P}w^{(p)}_0\prod_{s=1}^{t+1}\frac{\tilde{\varphi}_{s\to T}\left(\vartheta^{(p)}_{s-1\to T}\right)}{\tilde{\varphi}_{s-1\to T}\left(\vartheta^{(p)}_{s-1\to T}\right)}\right)^2}$$

$$= \frac{\mathbb{E}\left[\left(\prod_{s=1}^{t+1}\frac{\tilde{\varphi}_{s\to T}(\vartheta_{s-1\to T})}{\tilde{\varphi}_{s-1\to T}(\vartheta_{s-1\to T})}\right)^2\right]}{\left(\mathbb{E}\left[\prod_{s=1}^{t+1}\frac{\tilde{\varphi}_{s\to T}(\vartheta_{s-1\to T})}{\tilde{\varphi}_{s-1\to T}(\vartheta_{s-1\to T})}\right]\right)^2} = \rho_{t+1}.$$

The above equation implies that $P = \mathcal{O}(\rho_{t+1}(d_T))$ if we want to maintain an acceptable level for the ESS. In our context, even though the targets $(\bar{\varphi}_{s\to T})_s$ are d_T-dimensional the ratios of densities $(\varphi_{s\to T}/\varphi_{s-1\to T})_s$ will involve cancellations of "fill in" variables as discussed in the paper. This potentially leads to a much lower effective dimension of the problem than d_T.

For the SMC method presented in Dinh et al. (2018) in the context of phylogenetic trees, the authors have shown that ρ_T grows at most linearly in T under some strong conditions, somewhat comparable to the strong mixing conditions required in Theorem 1.2. Imposing an extra condition on the average branch length of the tree, ρ_T can be bounded uniformly in T. However, their method performs MH moves after resampling for improving the diversity of the particles, which could result in a sub-optimal algorithm. In contrast, TSMC uses MH moves for bridging φ_t and φ_{t+1} via the sequence of intermediate distributions $(\varphi_{t,k})_{k=1}^K$. Heuristically, the introduction of these intermediate distributions together with sensible transformations $\{G_{t\to t+1}\}$ should alleviate problems due to the dissimilarity of targets, thus providing control over ρ_T.

In this respect, the authors in Beskos et al. (2014) have analysed the stability of SMC samplers as the dimension of the state-space increases when the number of particles P is fixed. Their work provides justification, to some extent, for the use of intermediate distributions $(\varphi_{t,k})_{k=1}^{K}$. Under some assumptions, it has been shown that when the number of intermediate distributions $K = \mathcal{O}(d_T)$, and as $d_T \to \infty$, the effective sample size ESS_{t+1}^{P} is stable in the sense that it converges to a non-trivial random variable taking values in $(1, P)$. The total computational cost for bridging φ_t and φ_{t+1}, assuming a product form of d_T components, is $\mathcal{O}\left(Pd_T^2\right)$. Using this reasoning, we suspect TSMC will work well in similar and more complex scenarios, e.g. when the targets do not follow a product form or when strong mixing assumptions do not hold. This idea is supported by the results described in the paper.

2 Bayesian model comparison for mixtures of Gaussians

2.1 Split move

Suppose that at time t the transformation $G_{t\to t+1} : \Theta_t \times \mathcal{U}_t \to \Theta_{t+1} \times \mathcal{U}_{t+1}$ is selected from M_t possible candidates $\left\{G_{t\to t+1}^{(m)}\right\}_{m=1}^{M_t}$. The label of such transformation, denoted by l_t, is jointly drawn with u_t from the distribution $\psi_t(\cdot\,|\theta_t)$. Therefore, after sampling $(u_t, l_t) \sim \psi_t(\cdot\,|\theta_t)$, the incremental weight at t in the TSMC algorithm is given by

$$\frac{\tilde{\varphi}_{t+1}}{\tilde{\varphi}_{t\to t+1}}(\vartheta_{t\to t+1}) = \frac{\tilde{\pi}_{t+1}(\theta_{t\to t+1}(\vartheta_t))\,\psi_{t+1}(u_{t\to t+1}(\vartheta_t)|\,\theta_{t\to t+1}(\vartheta_t))}{\tilde{\pi}_t(\theta_t)\,\psi_t(u_t, l_t\,|\theta_t)\left|J_{t+1\to t}^{(l_t)}\right| M_t}, \tag{1}$$

where $J_{t+1\to t}^{(m)}$ denotes the Jacobian of $G_{t+1\to t}^{(m)}$. Notice that the denominator contains the term M_t since we have introduced the the extra variable L_t in the proposal; thus, in order to obtain the correct ratio of normalising constants we need to extend the target using a "dummy" distribution for L_t, in this case such distribution is uniform on the set $\{1, \ldots, M_t\}$.

The split move from Richardson and Green (1997) clearly falls into this category since the selected component to be split is chosen uniformly, i.e. $M_t = t$ and

$$\psi_t(u, l\,|\theta_t) = \frac{1}{t}\psi_t^{(s)}(u\,|\theta_t),$$

for $u \in \mathcal{U}_t$ and $l \in \{1, \ldots, t\}$; in this case $\psi_t^{(s)}$ is the distribution on the auxiliary variables U_t required for implementing the split move. An improvement on this idea would be to use a mixture representation of the proposal as done in Population Monte Carlo (Douc et al., 2007), i.e. the denominator of (1) would become

$$\varphi_{t\to t+1}(\vartheta_{t\to t+1}) = \pi_t(\theta_t)\sum_{l=1}^{M_t}\psi_t(u_t, l\,|\theta_t)\left|J_{t+1\to t}^{(l)}\right|; \tag{2}$$

however, we do not follow such approach. Instead, we try to alleviate a possible complication when implementing the split move. After selecting and splitting the k-th component (w_k, μ_k, τ_k), two new weights (say w_{k^-} and w_{k^+}), two new means (say μ_{k^-} and μ_{k^+}) and two new precisions (say τ_{k^-} and τ_{k^+}) are obtained. However, if either

$$\mu_{k^-} \notin [\mu_{k-1}, \mu_{k+1}] \qquad \text{or} \qquad \mu_{k^+} \notin [\mu_{k-1}, \mu_{k+1}],$$

then the incremental weight will be zero since the support of the target π_{t+1} has been restricted to ordered means. We solve this by reordering all the components with respect to their means and correcting the incremental weight with an extra factor. The correct incremental weight can be expressed as follows

$$\frac{\tilde{\varphi}_{t+1}}{\tilde{\varphi}_{t\to t+1}}(\vartheta_{t\to t+1}) = \frac{\tilde{\pi}_{t+1}(o_{t+1}(\theta_{t\to t+1}(\vartheta_t)))\,\psi_{t+1}(u_{t\to t+1}(\vartheta_t)|\,\theta_{t\to t+1}(\vartheta_t))}{\tilde{\pi}_t(\theta_t)\,\psi_t(u_t, l_t\,|\theta_t)\left|J_{t+1\to t}^{(l_t)}\right|\begin{pmatrix} t+1 \\ 2 \end{pmatrix}}, \tag{3}$$

where the function $o_{t+1} : \Theta_{t+1} \to \Theta_{t+1}$ simply combines the two newly created components, $(w_{k^-}, \mu_{k^-}, \tau_{k^-})$ and $(w_{k^+}, \mu_{k^+}, \tau_{k^+})$, with the set of already ordered $t-1$ components (those that were not split).

To see why (3) is correct we follow a similar reasoning for deriving (1). In order to obtain the correct ratio of normalising constants, we need to introduce a "dummy" distribution in the target. When inverting the split move

with rearrangement, two artificial variables are created denoting the labels of the newly created components. Since $\mu_{k^-} < \mu_{k^+}$, a simple choice for the "dummy" distribution is a uniform over the set

$$S_{t+1} = \{ (h,k) | \, h, k \in \{1, \dots, t+1\} \text{ and } h < k \} ,$$

for which $|S| = \begin{pmatrix} t+1 \\ 2 \end{pmatrix}$, as included in (3).

2.2 Birth move

The birth move can benefit also from a reordering of components. The correct incremental weight is much simpler than in the split case since the auxiliary variable $U_t \sim \psi_t^{(b)}(\cdot \,|\theta_t)$ already represents the new component (w_*, μ_*, τ_*). Using the same logic as before, when inverting the birth move with rearrangement an artificial variable is created which denotes the place of the most recent generated component. Since this label can take values in $S_{t+1} = \{1, \dots, t+1\}$, the simplest choice for the "dummy" distribution is a uniform over S_{t+1}; therefore, the expression for the incremental weight in this case is given by

$$\frac{\tilde{\varphi}_{t+1}}{\tilde{\varphi}_{t \to t+1}} (\vartheta_{t \to t+1}) = \frac{\tilde{\pi}_{t+1} \left(o_{t+1} \left(\theta_{t \to t+1} \left(\vartheta_t \right) \right) \right) \psi_{t+1} \left(u_{t \to t+1} \left(\vartheta_t \right) | \, \theta_{t \to t+1} \left(\vartheta_t \right) \right)}{\tilde{\pi}_t \left(\theta_t \right) \psi_t^{(b)} \left(u_t \,| \theta_t \right) |J_{t+1 \to t}| \, (t+1)} . \tag{4}$$

2.3 Marginalisation of moves

The previous descriptions of the birth and split moves are based on the idea of extending the target using an auxiliary distribution for the labels created due to the reordering process. We saw that a simple choice for this auxiliary distributions is a discrete uniform over the set of possible values for the labels, reason why the weights in (3) and (4) contain the denominator terms $\begin{pmatrix} t+1 \\ 2 \end{pmatrix}$ and $t+1$, respectively. However, as discussed later in the examples of Section 2.5, the corresponding estimators of the normalising constant may suffer from a very high variance making them useless from a practical point of view. A way around this problem is to marginalise the proposal over the artificial label created by the reordering process; such marginalisation is similar to (2) and is now described.

The ordering function $o_{t+1} : \Theta_{t+1} \to \Theta_{t+1}$, introduced previously, simply reorders the newly generated component (or components) from the birth (split) move. In order to be able to compute the inverse transformation of this reordering, an artificial variable $\bar{l}_{t+1} \in \bar{S}_{t+1}$ is created which simply denotes the place (or places) of the new component(s). To be more precise, there are two transformations applied to ϑ_t that allow us to obtain the final $\vartheta_{t \to t+1}$ together with the label $\bar{l}_{t+1}$. Let

$$\bar{G}_{t \to t+1}(\vartheta_t) := \bar{o}_{t+1} \circ G_{t \to t+1}(\vartheta_t) = \bar{o}_{t+1} \left(\theta_{t \to t+1} \left(\vartheta_t \right), u_{t \to t+1} \left(\vartheta_t \right) \right) = \left(o_{t+1} \left(\theta_{t \to t+1} \left(\vartheta_t \right) \right), u_{t \to t+1} \left(\vartheta_t \right), \bar{l}_{t+1} \right) = \left(\vartheta_{t \to t+1}, \bar{l}_{t+1} \right) ,$$

where $\bar{o}_{t+1} : \Theta_{t+1} \times \mathcal{U}_{t+1} \to \Theta_{t+1} \times \mathcal{U}_{t+1} \times \bar{S}_{t+1}$ is an extension of o_{t+1} that reorders $\theta_{t \to t+1}(\vartheta_t)$, leaves $u_{t \to t+1}(\vartheta_t)$ unchanged, and creates $\bar{l}_{t+1}$. In the previous sections there was no need to introduce $\bar{l}_{t+1}$ since the denominator in (3) and (4) is obtained simply by transforming back $\bar{\vartheta}_{t \to t+1}$ into ϑ_t; observe that for such cases

$$\varphi_{t \to t+1}(\vartheta_{t \to t+1}) = \varphi_t \left(\bar{G}_{t+1 \to t} \left(\vartheta_{t \to t+1}, \bar{l}_{t+1} \right) \right) |J_{t+1 \to t}| = \varphi_t \left(\vartheta_t \right) |J_{t+1 \to t}| \, .$$

The marginalisation step becomes clear by integrating out the variable $\bar{l}_{t+1}$; in this case the denominators in (3) and (4) respectively become

$$\varphi_{t \to t+1}(\vartheta_{t \to t+1}) = \sum_{\bar{l}_{t+1} \in \bar{S}_{t+1}} \varphi_t \left(\bar{G}_{t+1 \to t} \left(\vartheta_{t \to t+1}, \bar{l}_{t+1} \right) \right) \left| J_{t+1 \to t}^{(l_t)} \right| = \sum_{\bar{l}_{t+1} \in \bar{S}_{t+1}} \pi_t \left(\theta_t \right) \bar{\psi}_t \left(u_t, l_t \,| \theta_t \right) \left| J_{t+1 \to t}^{(l_t)} \right|$$

and

$$\varphi_{t \to t+1}(\vartheta_{t \to t+1}) = \sum_{\bar{l}_{t+1} \in \bar{S}_{t+1}} \varphi_t \left(\bar{G}_{t+1 \to t} \left(\vartheta_{t \to t+1}, \bar{l}_{t+1} \right) \right) |J_{t+1 \to t}| = \sum_{\bar{l}_{t+1} \in \bar{S}_{t+1}} \pi_t \left(\theta_t \right) \psi_t^{(b)} \left(u_t \,| \theta_t \right) |J_{t+1 \to t}| ,$$

recalling that the variables θ_t, u_t and l_t depend on $\left(\vartheta_{t \to t+1}, \bar{l}_{t+1} \right)$ via the inverse transformation $\bar{G}_{t+1 \to t}$.

In Section 2.5, we look at the performance of the marginalised versions of the birth and split moves against those described in Sections 2.1 and 2.2, which we term the conditional versions. It is clear that marginalising should be a sensible approach for reducing the variance of the estimated of the normalising constants, however in certain cases obtaining such marginal could become expensive or impractical if the required sum contains a large number of elements.

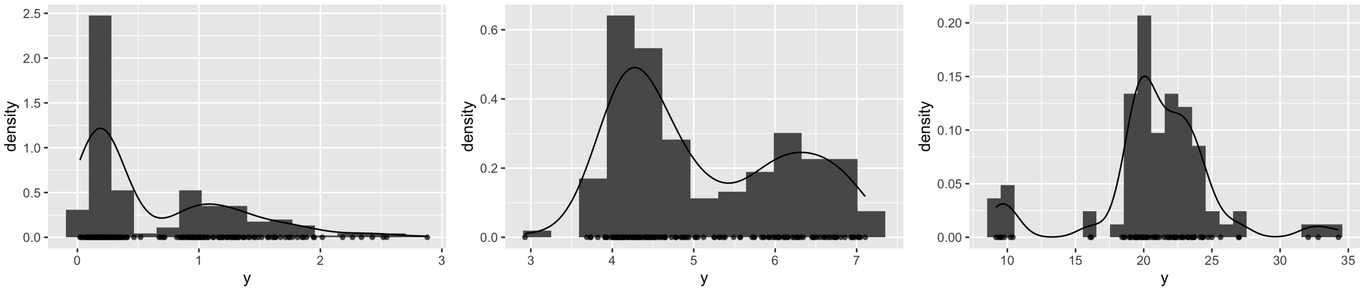

Figure 1: Density plots of the enzyme, acidity and galaxy data.

2.4 Details on the MCMC moves

The MCMC moves are performed in the transformed space of logit-weights, means and log-precisions. Given a set of t components $\{(w_k, \mu_k, \tau_k)\}_{k=1}^{t}$ at time t, the transformed components $\{(lw_k, \mu_k, l\tau_k)\}_{k=1}^{t}$ are given by

$$lw_k := \log\left(\frac{w_k}{w_t}\right) = \log\left(\frac{w_k}{1 - \sum_{j=1}^{t-1} w_j}\right),$$
$$l\tau_k := \log(\tau_k).$$

We consider two scenarios. For the first one we implement an adaptive Gaussian random-walk Metropolis algorithm on the transformed space, taking into account the Jacobian of the previous transformation. The adaptation is done in the proposal variance-covariance matrix in such way that the estimated acceptance probability from the particles stays near 0.20. More precisely, an initial diagonal variance-covariance matrix for the logit-weights, means and log-precisions is selected (say $\Sigma_{prop}^{(0)}$); then, after propagating the N particles, an estimated acceptance probability is obtained (say $\hat{\alpha}_P^{(0)}$). If such estimation lies outside a neighbourhood of 0.20, then a new matrix is obtained as follows

$$\Sigma_{prop}^{(1)} = \frac{\hat{\alpha}_P^{(0)}}{0.20}\Sigma_{prop}^{(0)}.$$

The process starts again (and is repeated until the desired acceptance probability is achieved) by propagating particles using $\Sigma_{prop}^{(1)}$ and computing the estimated acceptance $\hat{\alpha}_P^{(1)}$. One should be careful not to take a small number of particles or a small neighbourhood around 0.20 since the number of adaptations needed may be large. As seen in the following section, the previous choice of proposal could be quite inefficient since the particles may not move far from their current value. Nevertheless, using such an inefficient proposal will allow us to empirically quantify the effects of good and bad transformations $G_{t\to t+1}$: we present the results using this proposal in the following section.

For the second scenario (the one used in the results in the main body of the paper), using the set of particles approximately distributed according to $\varphi_{t\to t+1,k}$ we compute the empirical variance-covariance matrix $\hat{\Sigma}_{t+1,k}$. The proposal variance-covariance matrix for targeting $\varphi_{t\to t+1,k+1}$ is then chosen as follows $\Sigma_{prop} = \hat{\Sigma}_{t+1,k}/(t+1)$. This choice will certainly be a more sensible proposal provided a Gaussian proposal is able to capture the shape of the target and the consecutive intermediate distributions are similar; as seen in the following section, a carefully designed MCMC kernel (together with a good transformation $G_{t\to t+1}$) can dramatically improve the quality of the particles.

2.5 Results

This section shows results from SMC2 and the TSMC algorithms on the enzyme, acidity and galaxy data from Richardson and Green (1997) (see figure xxx). We ran the algorithms 50 times, up to a maximum of 8 components, with 500 particles. We used an adaptive sequence of intermediate distributions, choosing the next intermediate distribution to be the one the yields a CESS of βP, where $\beta = 0.99$. We resampled using stratified resampling when the ESS falls below αP, where $\alpha = 0.5$. The first adaptive MCMC scheme was used.

Figures 2, 3 and 4 show log marginal likelihood estimates from the different approaches, and the cumulative number of intermediate distributions used in estimating all of the marginal likelihoods up to model k for each k. The key observations from these results are:

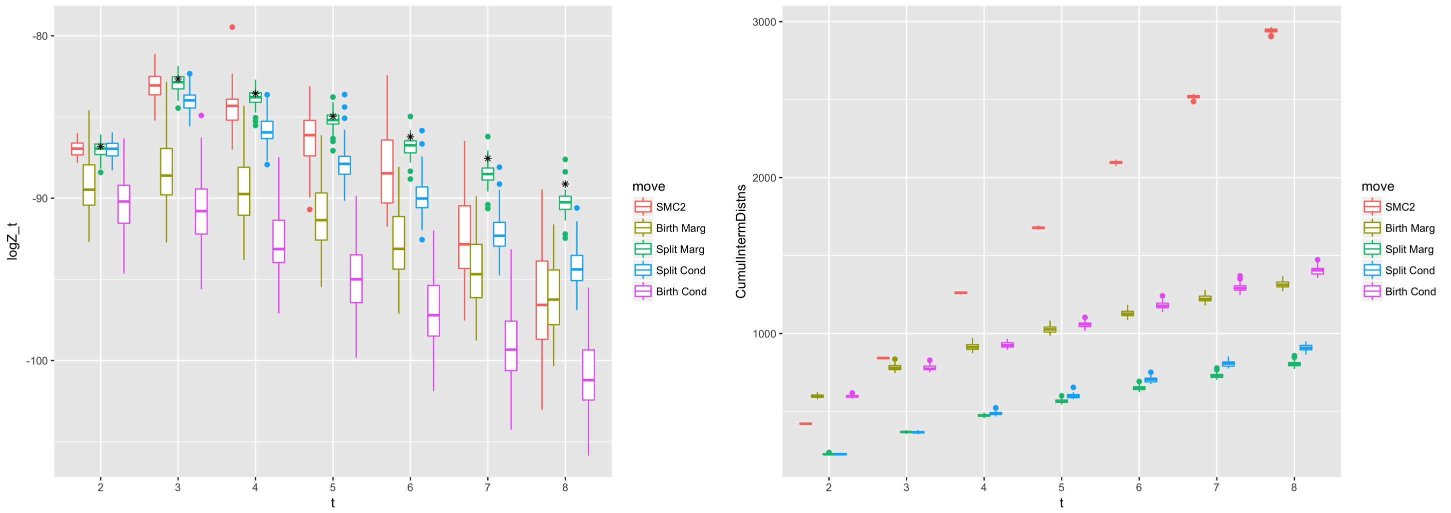

(a) Box plots of the log marginal likelihood estimates from each algorithm. Black dots represent the "truth" computed using a long SMC2 run. (b) The cumulative number of intermediate distributions up to model t.

Figure 2: The relative performance of the different SMC schemes on the enzyme data.

- The less effective adaptive MCMC scheme has a negative impact on the results (comparing figure 2 with those the in the main paper). Despite this, the marginal split TSMC still exhibits good performance on the enzyme and acidity data sets (in contrast to SMC2).
- TSMC appears to be less effective, compared to SMC2, on the galaxy data. When using the birth move, the reason is the same as stated in the main body of the paper: that the posterior on the parameters of existing components in model t does not provide a good proposal for these parameters in model $t+1$. For the split move, the results can be explained by the distribution of the data. This data set contains a few points located at a relatively large distance from the rest of the points in the dataset. The higher order models place components on these small clusters of points, whilst maintaining the most important components in the centre. In this case the split move is not an effective way to move between distributions (as also observed in Richardson and Green (1997)), thus the performance of TSMC with a split move is not as effective as SMC2, which uses the prior as the proposal for the parameters of all components.

3 Sequential Bayesian inference under the coalescent

3.1 Transformation and weight update

Let $g_t \sim \chi_t^{(g)}(\cdot \mid \theta_t, \mathcal{T}_t, y_{1:t+1})$ and $h_t^{(\text{new})} \sim \chi_t^{(h)}(\cdot \mid g_t, \theta_t, \mathcal{T}_t, , y_{1:t+1})$. The transformation $G_{t\to t+1}$ leaves θ, and $g_s, h_s^{(\text{new})}$ for $s > t$, unchanged. It makes a new tree from $\left(\mathcal{T}_t, g_t, h_t^{(\text{new})}\right)$ as follows. Firstly, g_t chooses a lineage to add the new branch to, where each possible lineage is indexed by the leaf on that lineage. Next we examine the coalescent heights. If ι is such that $h_t^{(\iota+1)} < h_t^{(\text{new})} < h_t^{(\iota)}$ then the effect of the transformation on the coalescence heights is

$$\left(\left(h_t^{(t)}, ..., h_t^{(2)}\right), h_t^{(\text{new})}\right) \mapsto \left(h_t^{(t)}, ..., h_t^{(\iota+1)}, h_t^{(\text{new})}, h_t^{(\iota)}, ..., h_t^{(2)}\right)$$

(or adding the new height to the beginning or the end of the vector if it is the first or last coalescence event), giving a Jacobian of 1. Then the new branching order is given by the original branching order, with a split in the branch that is uniquely determined by (ι, g_t), where the new branching order variable is denoted $b_t^{(\text{new})}$. We note that this transformation is not bijective: since branches higher up the tree are shared by multiple lineages there are multiple possible lineages that could have led to each tree.

Without loss of generality we examine the case of no intermediate distributions. As noted in the paper, $g_s, h_s^{(\text{new})}$ for $s > t$ are not involved in the weight update. The variables involved are $\mathcal{T}_{t+1}, \theta$, which have resulted from the

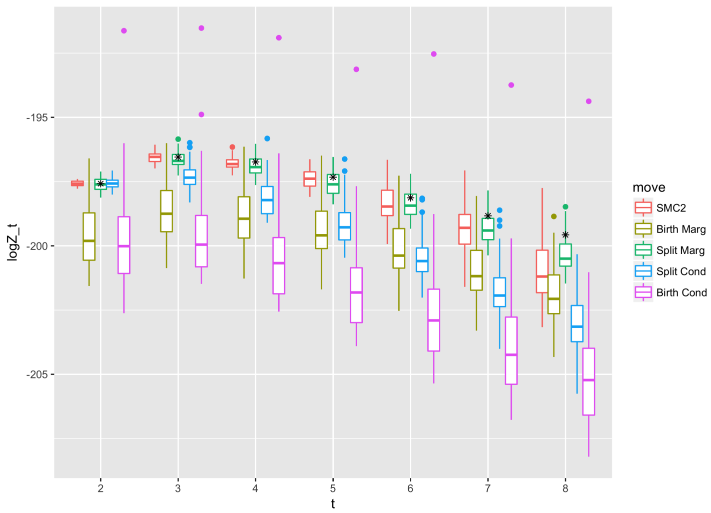

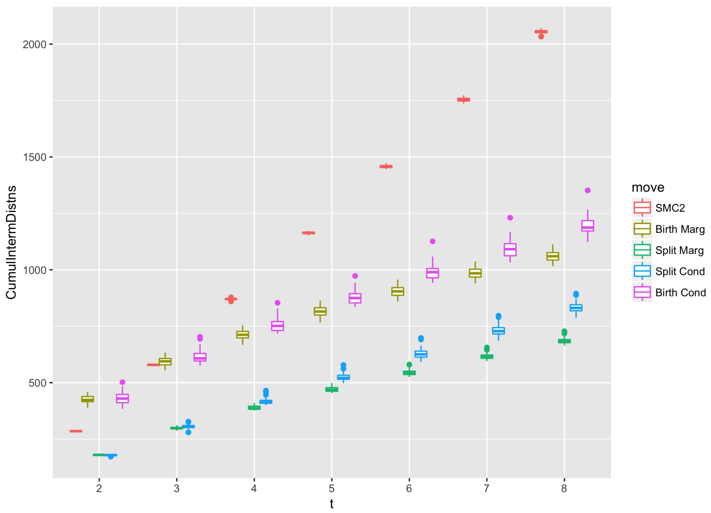

(a) Box plots of the log marginal likelihood estimates from each algorithm. Black dots represent the "truth" computed using a long SMC2 run.

(b) The cumulative number of intermediate distributions up to model t.

Figure 3: The relative performance of the different SMC schemes on the acidity data.

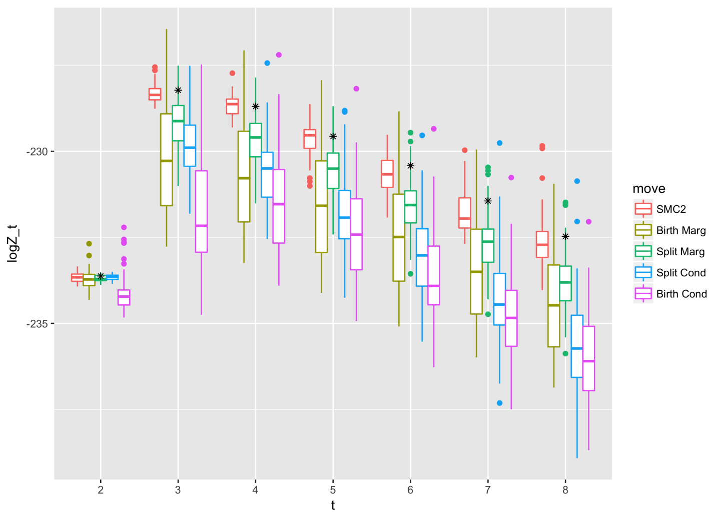

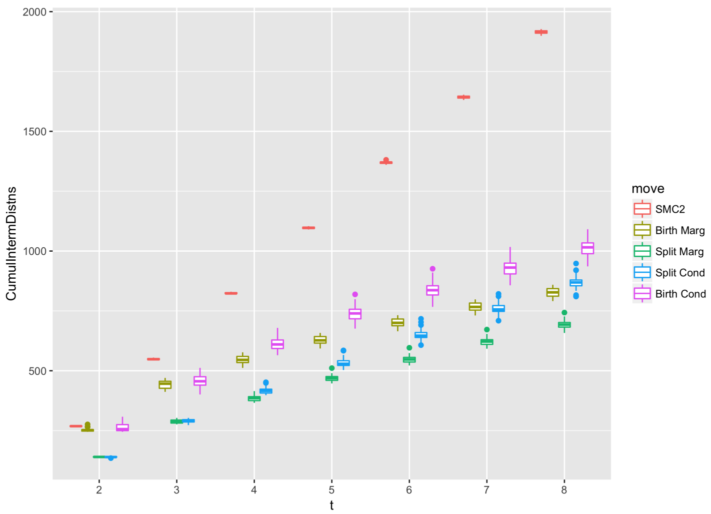

(a) Box plots of the log marginal likelihood estimates from each algorithm. Black dots represent the "truth" computed using a long SMC2 run.

(b) The cumulative number of intermediate distributions up to model t.

Figure 4: The relative performance of the different SMC schemes on the galaxy data.

application of $G_{t\to t+1}$. To find $\varphi_{t\to t+1}$ we must find the distribution under φ_t of the inverse image of $\mathcal{T}_{t+1}, \theta$. The resultant weight update is

$$\tilde{w}_{t+1} = w_t \frac{\pi_{t+1}\left(\mathcal{T}_{t+1}, \theta \mid y_{1:t+1}\right)}{\pi_t\left(\mathcal{T}_t, \theta \mid y_{1:t}\right)\left[\sum_{s\in\Lambda} \chi_t^{(g)}\left(g_t = s \mid \theta_t, \mathcal{T}_t, y_{1:t+1}\right) \chi_t^{(h)}\left(h_t^{(\mathrm{new})} \mid g_t = s, \theta_t, \mathcal{T}_t, y_{1:t+1}\right)\right]}, \tag{5}$$

where Λ is the set that contains the leaves of the lineages that could have resulted in $b_t^{(\mathrm{new})}$. Note the relationship with the Rao-Blackwellised weight update described in the paper: we achieve a lower variance through summing over the possible lineages rather than using an SMC over the joint space that includes the lineage variable.

3.2 Design of auxiliary distributions

For our SMC sampler to be efficient, we must design $\chi_t^{(g)}$ and $\chi_t^{(h)}$ such that the distributions in the numerator and denominator of (5) are close, i.e. resulting in many trees that have high probability under the posterior with $t+1$ sequences, but with the denominator having heavier tails than the numerator.

To choose the lineage, we make use of an approximation to the probability that the new sequence is M_s mutations from each of the existing leaves. Following Stephens and Donnelly (2000) (also see Li and Stephens, 2003) we choose the probability of choosing the lineage with leaf s using

$$\chi_t^{(g)}\left(s \mid \theta_t, y_{1:t+1}\right) \propto \left(\frac{N\theta_t}{t + N\theta_t}\right)^{M_s}. \tag{6}$$

This probability results from using a geometric distribution on the number of SNP differences between the new sequence and sequence s for each s, which is a generalisation of Ewens' sampling formula (Ewens, 1972) to the finite allele case. The geometric distribution results from integrating over possible coalescence times of the new sequence (where distribution on the time is modelled as exponentially distributed with the correct mean), yielding a choice for $\chi_t^{(g)}$ that is likely to give our importance sampling proposal a larger variance than our target.

For $\chi_t^{(h)}$ we propose to approximate the pairwise likelihood $f_{t+1,s}\left(y_s, y_{t+1} \mid \theta, h_t^{(\mathrm{new})}, g_t = s\right)$, where y_s is the sequence at the leaf of the chosen lineage. Since only two sequences are involved in this likelihood, it is likely to have heavier tails than the posterior. Our pairwise likelihood is

$$L\left(h_{\mathrm{t}}^{(new)} \mid y_s, y_{t+1}, \theta_t\right) = \left[\frac{3}{4} - \frac{3}{4}\exp\left(-4\theta_t h_{\mathrm{t}}^{(\mathrm{new})}/3\right)\right]^{M_s}\left[\frac{1}{4} + \frac{3}{4}\exp\left(-4\theta_t h_{\mathrm{t}}^{(\mathrm{new})}/3\right)\right]^{N-M_s},$$

where M_s is the number of pairwise SNP differences between the new sequence and sequence s, both of length N. This likelihood may be approximated by a distribution using the Laplace approximation $\mathcal{N}\left(\mu = \hat{h}, \sigma^2 = \left(-\hat{H}\right)^{-1}\right)$, where $\hat{h}_t^{(new)}$ denotes the maximum likelihood estimate of $h_t^{(\mathrm{new})}$ and $\hat{H}$ an estimate of the Hessian of the log likelihood at this estimate (Bishop, 2006). Reis and Yang (2011) proposes an accurate approximation of the two sequence likelihood by using a Laplace approximation in a transformed space, in particular they propose to use the transformation $2\arcsin\sqrt{\frac{3}{4} - \frac{3}{4}\exp\left(-2\theta_t h_t^{(\mathrm{new})}/3\right)}$. In this case the mean and variance of the Gaussian approximation are respectively $\mu = \hat{h}$ and $\sigma^2 = \left(-\hat{H}\right)^{-1}$ where $\hat{h} = 2\arcsin\sqrt{\frac{M_s}{N}}$ and $\hat{H} = -N$. Thus in order to simulate a new height $h^{(\mathrm{new})}$ we first simulate $\beta \sim \mathcal{N}\left(2\arcsin\sqrt{\frac{M_s}{N}}, 1/N\right)$ and then compute

$$h_t^{(\mathrm{new})} = -\frac{3}{4\theta_t}\log\left(1 - \frac{4}{3}\sin^2\left(\beta/2\right)\right). \tag{7}$$

The density of this distribution is given by

$$\chi_t^{(h)}\left(h_t^{(\text{new})}\right) = \left|\frac{2\theta\exp\left\{-\frac{4}{3}\theta h_t^{(\text{new})}\right\}}{\sqrt{3}\sqrt{1-\frac{3\left(1-\exp\left\{-\frac{4}{3}\theta h_t^{(\text{new})}\right\}\right)}{4}}\sqrt{1-\exp\left\{-\frac{4}{3}\theta h_t^{(\text{new})}\right\}}}\right| \times\frac{1}{\sigma\sqrt{2\pi}}\exp\left(-\frac{\left(2\arcsin\sqrt{\frac{3}{4}\left(1-\exp\left\{-\frac{4}{3}\theta h_t^{(\text{new})}\right\}\right)}-\mu\right)^2}{2\sigma^2}\right).$$

3.3 SMC and MCMC details

Our MCMC moves when moving from target t to $t+1$ are technically made on the space E_{t+1}, and in practice made on the space Θ_{t+1} (recalling that the variables $u_{t+1\to t}$ will be updated by direct simulation from ψ_{t+1}). The default configuration of our method was as follows. We use the following moves on each parameter in Θ_{t+1}: for θ we use a multiplicative random walk, i.e. an additive normal random walk in log-space, with proposal variance σ_θ^2 in log-space; for each height $h^{(a)}$ $(2 < a < T+1)$ we use a truncated normal proposal with mean the current value of $h^{(a)}$ and variance $\sigma^2_{h^{(a)}}$. For the branching order we use 20 subtree pruning and regrafting (SPR) moves in each sweep of the MCMC: pilot runs suggested that this many proposed moves result in approximately 0.5 moves being accepted at each sweep of the MCMC; adaptive methods may also be used to make such a choice automatically (South et al., 2019).

The SMC uses $P = 250$ particles, with an adaptive sequence of intermediate distributions, choosing the next intermediate distribution to be the one the yields a CESS of βP, where $\beta = 0.95$. We used stratified resampling when the ESS falls below αP, where $\alpha = 0.5$. At each iteration we used the current population of particles to tune the proposal variances σ_θ^2 and $\sigma^2_{h^{(a)}}$ for each a. Each variance was decomposed into two terms as follows $\sigma^2 = s\hat{\sigma^2}$, with $\hat{\sigma^2}$ being an empirical variance and s being a scaling factor (different for each proposal variance). $\hat{\sigma^2_\theta}$ was taken to be the empirical variance of the weighted particles for θ. $\hat{\sigma^2_{h^{(a)}}}$ was taken to be the empirical variance of the residuals after using a linear regression of $h^{(a)}$ on θ (used due to the strong dependence of $h^{(a)}$ on θ). Each scaling s was initialised to 1, and: doubled at each iteration where the acceptance rate was estimated as greater than 0.6; halved where the rate was estimated at less than 0.15.

References

S. Agapiou, O. Papaspiliopoulos, D. Sanz-Alonso, and A. M. Stuart. Importance sampling: Intrinsic dimension and computational cost. *Statist. Sci.*, 32(3):405–431, 08 2017. doi: 10.1214/17-STS611.

A. Beskos, D. Crisan, and A. Jasra. On the Stability of Sequential Monte Carlo Methods in High Dimensions. *The Annals of Applied Probability*, 24(4):1396–1445, 2014.

P. Bickel, B. Li, and T. Bengtsson. Sharp failure rates for the bootstrap particle filter in high dimensions. In *Pushing the Limits of Contemporary Statistics: Contributions in Honor of Jayanta K. Ghosh*, volume 3, pages 318–329, 2008.

C. M. Bishop. *Pattern Recognition and Machine Learning.* Springer, 2006. ISBN 9780387310732.

F. Cérou, P. Del Moral, and A. Guyader. A nonasymptotic theorem for unnormalized Feynman–Kac particle models. *Annales de l'Institut Henri Poincaré*, 47(3):629–649, 2011.

N. Chopin. Central limit theorem for sequential Monte Carlo methods and its application to Bayesian inference. *The Annals of Statistics*, 32(6):2385–2411, 2004. ISSN 0090-5364.

P. Del Moral. *Feynman-Kac Formulae.* Springer, 2004.

P. Del Moral, A. Doucet, and A. Jasra. Sequential Monte Carlo samplers. *Journal of the Royal Statistical Society: Series B*, 68(3):411–436, 2006.

V. Dinh, A. E. Darling, and F. A. Matsen IV. Online bayesian phylogenetic inference: Theoretical foundations via sequential monte carlo. *Systematic Biology*, 67(3):503–517, 2018.

R. Douc, A. Guillin, J. M. Marin, and C. P. Robert. Convergence of adaptive mixtures of importance sampling schemes. *The Annals of Statistics*, 35(1):420–448, 2007. ISSN 0090-5364.

W. J. Ewens. The sampling theory of selectively neutral alleles. *Theoretical Population Biology*, 3:87–112, 1972.

N. Li and M. Stephens. Modeling Linkage Disequilibrium and Identifying Recombination Hotspots Using Single-Nucleotide Polymorphism Data. *Genetics*, 165:2213–2233, 2003.

M. Reis and Z. Yang. Approximate Likelihood Calculation on a Phylogeny for Bayesian Estimation of Divergence Times. *Molecular Biology and Evolution*, 28(1969):2161–2172, 2011.

S. Richardson and P. J. Green. On Bayesian Analysis of Mixtures with an Unknown Number of Components (with discussion). *Journal of the Royal Statistical Society: Series B (Statistical Methodology)*, 59(4):731–792, 1997. ISSN 1369-7412.

L. F. South, A. N. Pettitt, and C. C. Drovandi. Sequential monte carlo samplers with independent markov chain monte carlo proposals. *Bayesian Anal.*, 14(3):753–776, 09 2019.

M. Stephens and P. Donnelly. Inference in molecular population genetics. *Journal of the Royal Statistical Society Series B*, 62(4):605–655, 2000.
